# Identification and Characterization of *msf*, a Novel Virulence Factor in *Haemophilus influenzae*

**DOI:** 10.1371/journal.pone.0149891

**Published:** 2016-03-15

**Authors:** Jennifer M. Kress-Bennett, N. Luisa Hiller, Rory A. Eutsey, Evan Powell, Mark J. Longwell, Todd Hillman, Tenisha Blackwell, Barbara Byers, Joshua C. Mell, J. Christopher Post, Fen Z. Hu, Garth D. Ehrlich, Benjamin A. Janto

**Affiliations:** 1 Center for Genomic Sciences, Allegheny Singer Research Institute, Allegheny General Hospital, Pittsburgh, Pennsylvania, United States of America; 2 Department of Microbiology and Immunology, Drexel University College of Medicine, Philadelphia, Pennsylvania, United States of America; 3 Center for Genomic Sciences, Institute for Molecular Medicine and Infectious Disease, Drexel University College of Medicine, Philadelphia, Pennsylvania, United States of America; 4 Department of Biological Sciences, Carnegie Mellon University, Pittsburgh, Pennsylvania, United States of America; 5 Department of Otolaryngology, Head and Neck Surgery, Drexel University College of Medicine, Allegheny Campus, Pittsburgh, Pennsylvania, United States of America; East Carolina University School of Medicine, UNITED STATES

## Abstract

*Haemophilus influenzae* is an opportunistic pathogen. The emergence of virulent, non-typeable strains (NTHi) emphasizes the importance of developing new interventional targets. We screened the NTHi supragenome for genes encoding surface-exposed proteins suggestive of immune evasion, identifying a large family containing Sel1-like repeats (SLRs). Clustering identified ten SLR-containing gene subfamilies, each with various numbers of SLRs per gene. Individual strains also had varying numbers of SLR-containing genes from one or more of the subfamilies. Statistical genetic analyses of gene possession among 210 NTHi strains typed as either disease or carriage found a significant association between possession of the SlrVA subfamily (which we have termed, macrophage survival factor, *msf*) and the disease isolates. The PittII strain contains four chromosomally contiguous *msf* genes. Deleting all four of these genes (*msfA1-4*) (KO) resulted in a highly significant decrease in phagocytosis and survival in macrophages; which was fully complemented by a single copy of the *msfA1* gene. Using the chinchilla model of otitis media and invasive disease, the KO strain displayed a significant decrease in fitness compared to the WT in co-infections; and in single infections, the KO lost its ability to invade the brain. The singly complemented strain showed only a partial ability to compete with the WT suggesting gene dosage is important *in vivo*. The transcriptional profiles of the KO and WT in planktonic growth were compared using the NTHi supragenome array, which revealed highly significant changes in the expression of operons involved in virulence and anaerobiosis. These findings demonstrate that the *msfA1-4* genes are virulence factors for phagocytosis, persistence, and trafficking to non-mucosal sites.

## Introduction

The impact of the gram-negative coccobacillus, *Haemophilus influenzae* on human health has changed significantly over the past several decades, and may continue to do so in the future. Prior to routine immunization against the highly virulent serotype b form (Hib), *H*. *influenzae* was a leading cause of pediatric bacterial meningitis and epiglottitis in the United States [1]. In the post-vaccine era the NTHi are opportunistic pathogens causing and exacerbating multiple upper and lower respiratory tract illnesses including otitis media (OM) [2–4], otorrhea [5, 6], sinusitis [7], bronchitis and chronic obstructive pulmonary disease (COPD) [8], pneumonia [9], and conjunctivitis [10]. Furthermore, the NTHi are early colonizers of the lungs of children with cystic fibrosis, suggesting they may play a critical role in the bacterial pathogenesis of this disease by causing damage that allows for infection by more virulent pathogens such as *Pseudomonas aeruginosa*. Despite a marked reduction in overall *H*. *influenzae* invasive disease post-vaccine, non-typeable strains continue to cause invasive complications such as meningitis and bacteremia albeit with low incidence. Worryingly, several studies in various post-vaccine populations have observed steadily increasing NTHi invasive incidence rates, highlighting the importance of investigating the mechanisms of invasiveness in the absence of capsule [11–16].

Comparison of the whole genome sequences (WGS) of multiple *H*. *influenzae* isolates has revealed enormous genomic diversity among strains [17–19]. In addition to extensive single-nucleotide polymorphisms, the species as a whole contains many more distributed/accessory genes than core genes, *i*.*e*. genes present in only a subset of isolates versus those present in all isolates. Thus the species-level supragenome (or “pan-genome”) is several times the size of the genome of a single strain. On average, each strain-pair differs by the possession of ~ 400 genes [17]. These extensive differences in gene possession among strains lead to profoundly different phenotypes with respect to disease causation [20]. This diversity in genotype and phenotype is consistent with a role for individual genes in virulence, and suggests that effective prevention and treatment strategies will develop from specifically targeting distributed/accessory virulence determinants [21, 22]. Rational vaccine design currently focuses on highly expressed, surface-exposed core gene products ensuring broad coverage of entire bacterial species [23]. While this is desirable for human pathogens that are not part of the normal microflora, targeting core genes of opportunistic pathogens results in the eradication of the commensal populations as well as the disease causing populations of the target species. Thus, conceptually, this is akin to treatment with antibiotics, which results in major disruptions to the hosts' normal microbiota leading to further acute and chronic conditions [24–27]. In these cases we propose that alternative “microbiome-sparing” approaches should be investigated. In this way, carriage strains lacking virulence genes could be spared, leaving the host’s commensal ecosystem intact.

Multiple surface molecules have been associated with pathogenesis and immunity in both acute and chronic NTHi disease. The ability to bind to various cell types depends on adhesins such as Hif, Hmw1/2, Hap, Hia/Hsf, OMP-2,5, oapA and PCP [28, 29]. These gene products are frequently under phase variable control due to tandem repeats within or slightly upstream of the coding sequences. The coding sequences are also highly variable from strain to strain and rife with repetitive sequences, which accumulate as a result of immune pressure on these surface exposed molecules [30–33]. Other surface molecules play an important role in *Haemophilus* virulence, in particular, the lipo-oligosaccharide (LOS). Secondary modification of the LOS results in considerable antigenic heterogeneity among and within strains and is driven again by phase variable genes such as *lic*, *lgt*, *lsg*, and *sia* genes [34, 35]. Individual strains often do not possess all of these virulence factors; they possess only a subset of them. Thus it has been proposed that possession of certain subsets provides fitness advantages in different settings (middle ear, lung, nasopharynx etc.) [22].

NTHi have been observed within host cells in *in vitro* and *in vivo* assays as well as in clinical tissue samples suggesting that survival and persistence within host cells plays a role in chronic disease [36–49]. There is also a correlation between the ability of bacteria to survive in macrophages and disease outcome or severity. In the rat model, strains able to survive in macrophages *in-vitro* had an increased ability to cause systemic disease [50]. Similar observations have been made in several other bacterial species [51–54].

Here we present an initial characterization of Msf, a novel distributed NTHi virulence factor with a role in macrophage survival and disease.

## Results

### Identification of a novel protein family in *H*. *influenzae*, characterized by variable numbers of SLR motifs per protein, and variable numbers of genes per strain

**Fig 1:**
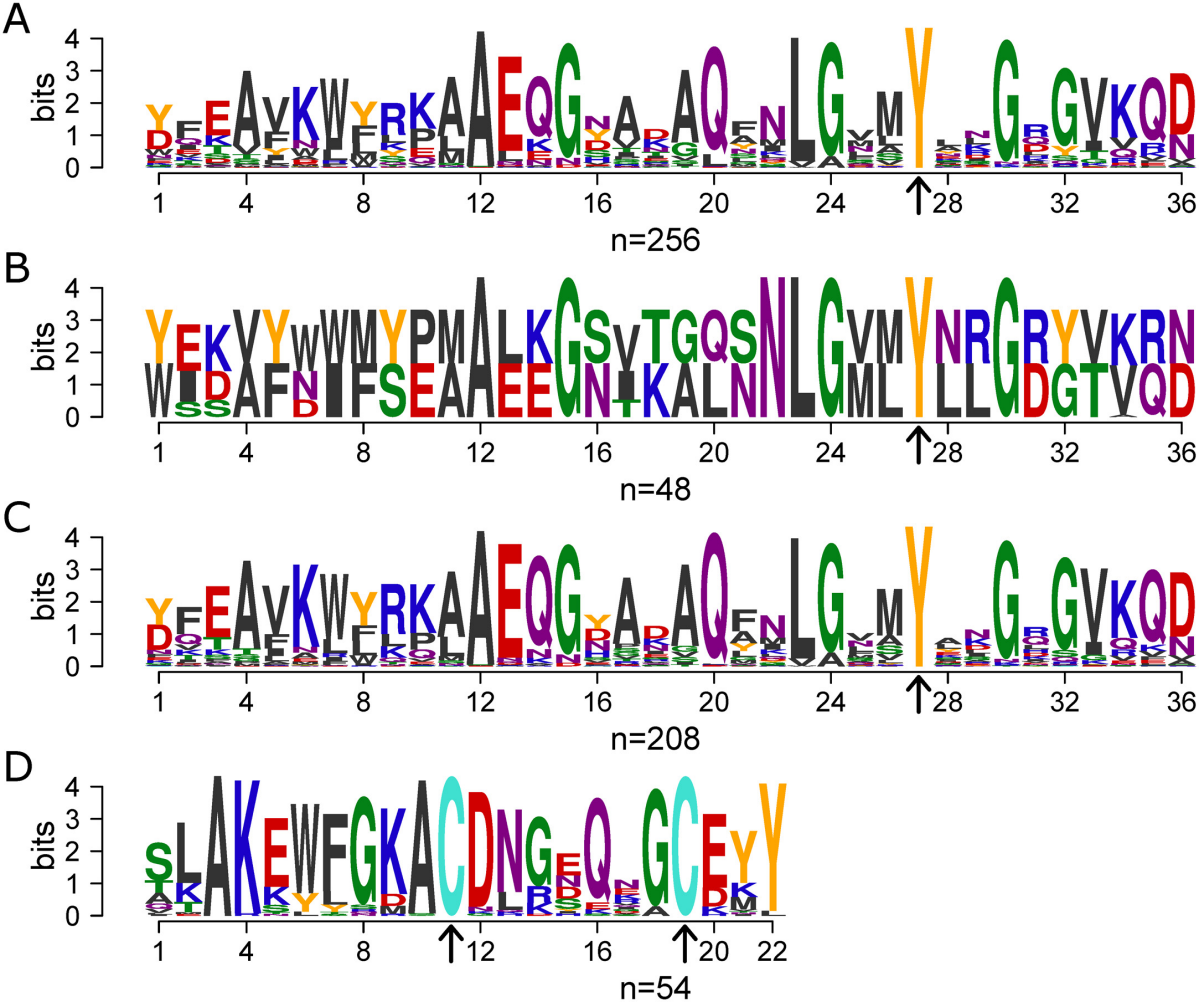
The Sel1-like Repeat motif found in *H*. *influenzae*. Sequence logos based on MEME/MAST analysis that represent various motifs found in SLR genes. Motifs were generated using the R Bioconductor package motifStack. Amino acid colors are a modification of the WebLogo default, with Tyr and Cys having unique colors (Y = orange and C = turquoise). **(A-C)** Arrows indicate the location of the 100% conserved tyrosine residue. **(A)** SLR consensus motif found among all 79 SLR-containing genes (256 motifs) illustrating conserved alanine and glycine residues. The only 100% conserved residue is the tyrosine residue at position 27. **(B)** SLR consensus motif found in the 24 *slrC* genes (48 motifs). **(C)** SLR consensus motif found in all 55 *slrV* genes (208 motifs). **(D)** Consensus motif found in the C-terminus of all but one *slrV* gene which contain equidistant cysteine residues (arrows) (54 motifs).

High genetic diversity, variation in repetitive motifs as well as multiple gene copies with allelic differences are all associated with immune evasion, and these are also common characteristics of virulence determinants such as adhesins, autotransporters and other host-interacting proteins [22, 30–35, 55–57]. We analyzed the *H*. *influenzae* supragenome developed from whole genome sequencing (WGS) of 24 geographically and clinically diverse isolates (Table 1) for proteins that fit these criteria. 47,997 open reading frames (ORFs) were identified and were grouped into 3100 orthologous gene clusters by virtue of their sharing at least 70% amino acid sequence identity over 70% of the length [17, 19]. Manual curation of these gene clusters revealed a large set of genes, distributed among several clusters, that all contained the Sel1 Pfam motif (PF08238). From an initial list of genes identified to contain this motif, we performed multiple iterations of MEME/MAST on the *H*. *influenzae* supragenome [58–60]. MEME analysis identified the consensus Sel1-like repeat (SLR) motif shared among these ORFs. MAST was then used to iteratively search the entire 24-strain supragenome for new instances of this repeat in the already identified ORFs, as well as new ORFs containing it. This analysis combined with manual curation identified a total of 79 ORFs, which were represented by 10 supragenome gene clusters (S1 Table). The common SLR motif identified by MEME/MAST is 36 amino acid residues long and is characterized by conserved alanine and glycine amino acids, as well as a 100% conserved tyrosine residue (Fig 1A).

**Table 1:** Clinical and geographic origins of 24 whole genome sequenced strains.

Strain	# of gene clusters	Clinical origin	Geographic origin	GenBank BioProject	Reference
3655	1991	Blood	San Diego, CA	PRJNA54385	[121]
22.1–21	1967	Nasopharynx	Ann Arbor, MI	PRJNA16398	[122]
22.1–24	1973	Nasopharynx	Ann Arbor, MI	PRJNA29373	[122]
22.4–21	1957	Nasopharynx	Ann Arbor, MI	PRJNA16396	[122]
6P18H1	2015	COPD	Iowa City, Iowa	PRJNA55127	[19]
7P49H1	1897	COPD	Buffalo, NY	PRJNA55129	[19]
86-028NP	2001	Nasopharynx	Nationwide Children’s Hospital, Columbus, OH	PRJNA58093	[115]
B10810	2044	Meningitis	United Kingdom	PRJNA86647	Wellcome Trust Sanger Institute
NML20	1838	Blood	Manitoba, Canada	PRJNA29375	[19]
PittAA	2003	COME, tube replacement	Children’s Hospital, Pittsburgh, PA	PRJNA54391	[123]
PittBB	1922	COME, tube replacement	Children’s Hospital, Pittsburgh, PA	PRJNA16402	[19]
PittCC	1915	COME, tube replacement	Children’s Hospital, Pittsburgh, PA	PRJNA18099	[19]
PittDD	1865	COME, tube replacement	Children’s Hospital, Pittsburgh, PA	PRJNA16392	[19]
PittEE	1848	COME, tube replacement	Children’s Hospital, Pittsburgh, PA	PRJNA58591	[123]
PittGG	1966	AOM, ottorhea	Children’s Hospital, Pittsburgh, PA	PRJNA58593	[123]
PittHH	1948	COME, tube replacement	Children’s Hospital, Pittsburgh, PA	PRJNA54393	[123]
PittII	2057	AOM, ottorhea	Children’s Hospital, Pittsburgh, PA	PRJNA54395	[123]
PittJJ	2054	COME, tube replacement	Children’s Hospital, Pittsburgh, PA	PRJNA18103	[19]
R1838	1900	Blood	Papua New Guinea	PRJNA29377	[19]
R2846	1856	OME	Seattle, WA	PRJNA161921	[124, 125]
R2866	2017	Blood	Seattle, WA	PRJNA161923	[124, 126]
R3021	2017	Nasopharynx	Seattle, WA	PRJNA54397	[124]
R393	2109	Sputum isolate	Malaysia	PRJNA29379	[19]
Rd KW20	1892	Laboratory Strain	Columbia University	PRJNA57771	[127]

COME—Chronic otitis media with effusion; AOM—Acute otitis media; COPD—Chronic obstructive pulmonary disease

Sequences in one of the ten SLR-containing gene clusters are highly similar to each other (at least 95% amino acid identity over 95% of their length), and are present at exactly one copy in each of the 24 strains at a common genomic locus (Table 2). This single, core SLR gene contains an N-terminal signal peptide sequence and two tandem SLR motifs that differ in sequence (Fig 1B). We refer to this gene cluster as the core SLR (SlrC) subfamily.

**Table 2:** Number of SLR-containing genes found at each SLR Locus among 24 whole genome sequenced strains.

Strain	Core Locus	SlrV Locus 1	SlrV Locus 2	SlrV Locus 3	SlrV Locus 4
3655	1	0	0	0	2
22.1–21	1	2	0	0	0
22.1–24	1	2	0	0	0
22.4–21	1	0	0	0	0
6P18H1	1	0	0	0	0
7P49H1	1	3	0	0	0
86-028NP	1	4	0	0	0
B10810	1	0	0	0	0
NML20	1	3	0	0	0
PittAA	1	0	0	0	0
PittBB	1	3	0	0	0
PittCC	1	2	0	0	0
PittDD	1	2	0	0	0
PittEE	1	3	0	0	0
PittGG	1	3	2	0	0
PittHH	1	4	0	0	0
PittII	1	4	0	0	0
PittJJ	1	2	0	0	0
R1838	1	4	0	1	0
R2846	1	3	0	0	0
R2866	1	4	0	0	0
R3021	1	0	0	0	0
R393	1	2	0	0	0
Rd KW20	1	0	0	0	0

**Fig 2:**
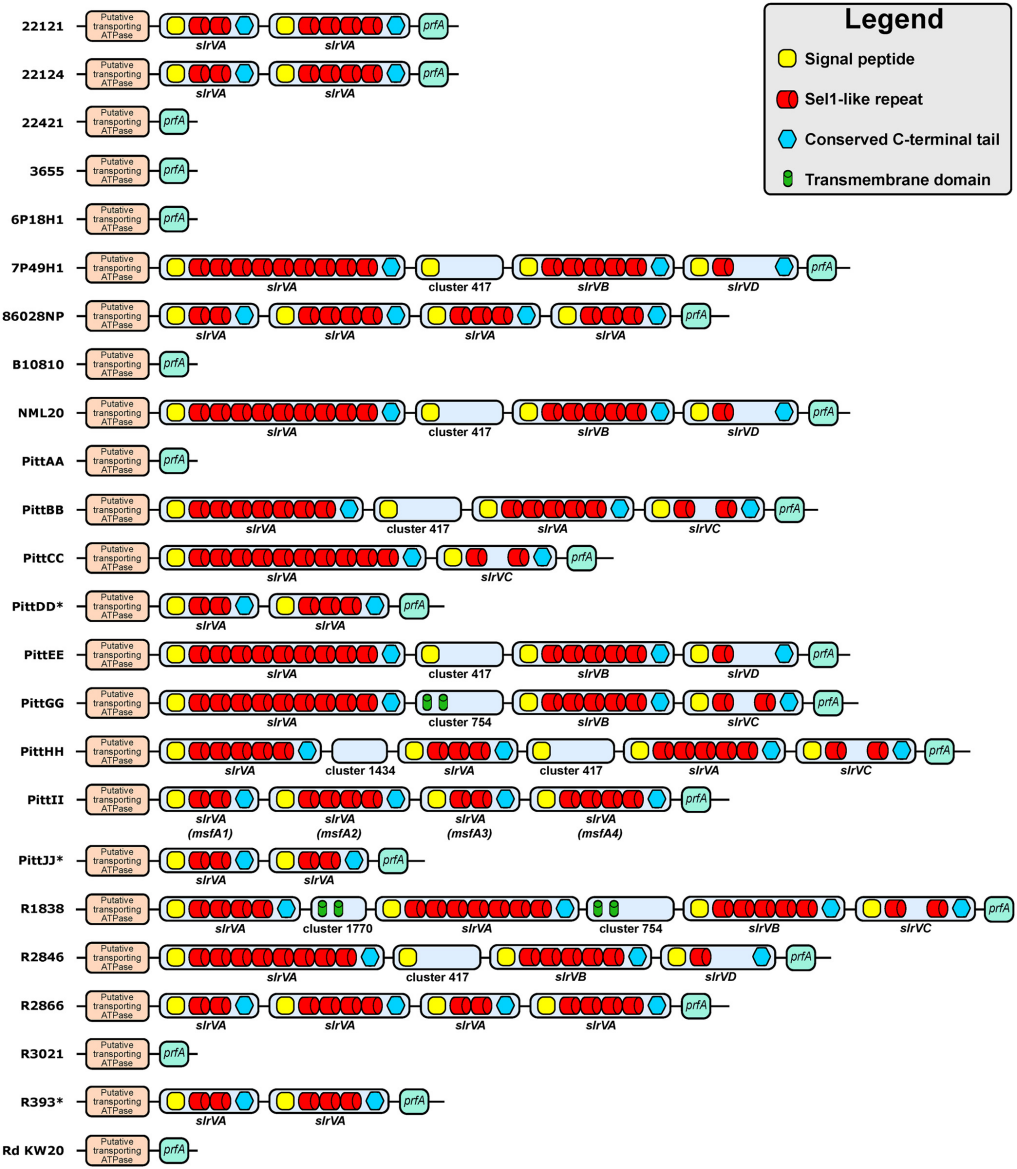
Chromosomal schematic of SlrV locus 1 and architecture of the *slrV* genes in 24 sequenced *H*. *influenzae* strains. SlrV locus 1 is located between core genes encoding a putative transporting ATPase and peptide chain release factor 1 (*prfA*). Strain PittII contains four *slrVA* genes in tandem; two with 2 SLR and two with 4 SLR. These genes correspond to *msfA1-4* in this manuscript. Only genes predicted to encode full-length products are illustrated. 7 genomes do not contain any full-length *slrV* gene at this locus. * denotes genomes in which SlrV locus 1 is located on the edges of contig breaks. Therefore it is possible that there are more *slrV* genes or SLR motifs located in the assembly gaps.

The remaining 55 SLR-containing ORFs represented by nine distributed gene clusters were collectively named the variable SLR (SlrV) subfamily (Fig 1C shows the consensus SLR motif found in the SlrV proteins). The individual SlrV subfamilies are referred to as SlrVA-I and are almost always found in tandem at a second non-core genomic locus described below. Similar to the SlrC, all SlrV proteins also contain an N-terminal signal peptide predicting that they are secreted or membrane associated. Each SlrV subfamily has unique variations of the SLR motif sequence (S1 Fig, S2 Table). However, conservation of several key residues can be seen within all SlrV, including a 100% conserved tyrosine at position 27. In addition, almost all SlrV (but not SlrC) contain a conserved C-terminus with two equi-distant cysteine residues (Fig 1D). Individual strains vary with respect to the total number of *slrV* genes, ranging from 0 to 5 (Table 2), and as to whether or not they contain genes from multiple SlrV subfamilies (Fig 2). Strains with multiple copies from the same SlrV subfamily can also display heterogeneity with respect to the number of motif repeats per gene (Fig 2, S3 Table).

**Fig 3:**
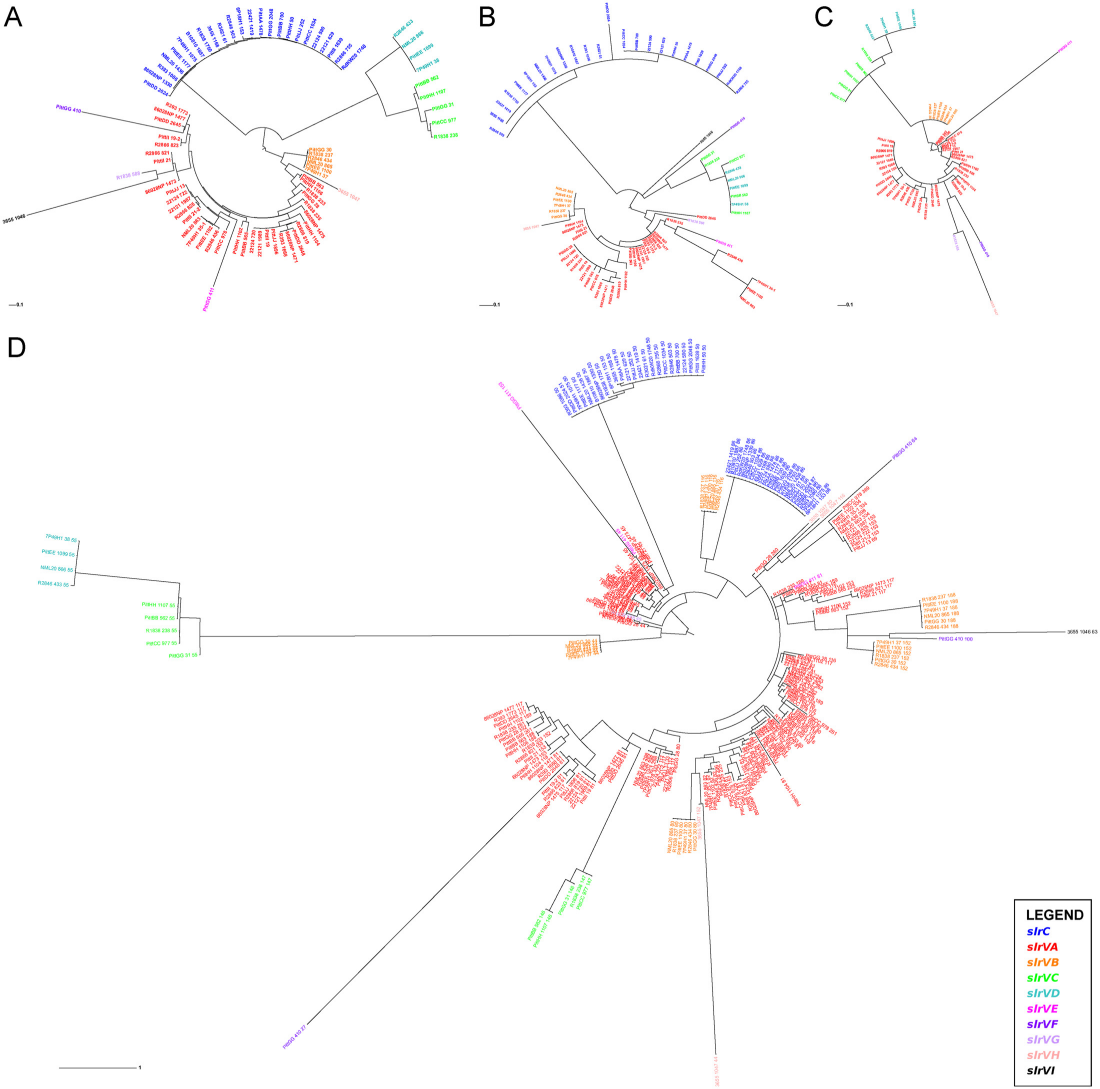
Phylogenetic inference from SLR genes and protein domains. Maximum-likelihood trees were calculated using RAxML [111] and visualized with the interactive Tree of Life web server (http://itol.embl.de) [112, 113]. Colored nodes represent the SLR subfamily from which the particular sequence was extracted from (see Legend). **(A)** Entire SLR gene sequences (n = 79) demonstrating the justification for clustering into different subfamilies. Node labels indicate the strain and strain-specific SGH cluster ID number. **(B)** 20–26 amino acid long signal peptides (n = 78) from each SLR protein. For the most part the tree structure reflects that of the whole gene tree with individual SLR subfamilies clustering together. Node labels indicate the strain and strain-specific SGH cluster ID number. **(C)** 22 amino acid C-terminal motif that was identified in 54 SlrV proteins. SlrC proteins do not contain this motif and thus are not included in the analysis. Again, individual SlrV subfamilies cluster together. Node labels indicate the strain and strain-specific SGH cluster ID number. **(D)** 36 amino acid SLR motifs extracted from the 79 genes containing them (n = 256). Motifs found within the same protein do not cluster together. Node labels indicate the strain, strain-specific SGH cluster ID number, and location of the motif within the CDS (amino acid position). S3 Fig presents a rectangular version of this tree without text overlap.

Due to the original clustering requirements we expect that each SLR gene family has a distinct biological function that may or may not be related. This is supported by the fact that the different SLR subfamilies can be easily distinguished by examining the sequences of their signal peptides and C-termini. Phylogenetic trees generated from entire SLR gene sequences (Fig 3A) closely resemble those based on just the signal peptides (Fig 3B) or C-termini (Fig 3C) from those genes. However, individual motifs do not cluster in the same manner. Motifs located within the same protein (and thus of the same SLR subfamily) are dispersed around the tree, often clustering more closely with motifs found in other proteins (often in other SLR subfamilies) (Fig 3D). This is illustrated best by the five SLR motifs found in tandem in each *slrVB* gene, two of which are closely related by sequence, one which is more closely related to *slrC* motifs and two of which are more closely related with *slrVA* motifs. This reveals a complicated evolutionary history in the SLR-containing genes likely driven by gene and motif duplication/contraction, as well as horizontal gene transfer in this naturally transformable organism.

### Organization of *slrV* genes on the chromosome

The vast majority (50/55) of the *slrV* genes are found at a common chromosomal locus (SlrV locus 1). It is flanked by a putative transporting ATPase (COG3101) and peptide chain release factor 1 (*prfA*) (Fig 2). The SlrVA subfamily accounts for over 60% of the total *slrV* genes and is always found at this locus. When present, *slrVB*, *slrVC* and *slrVD* are also found at this locus. In most of the genomes that do not have any genes present at SlrV locus 1 (22421, 3655, 6P18H1, PittAA, R3021, B10810, and RdKW20), remnants of *slrVC* or *slrVD* can be found, suggesting that these genes are ancestral and have been lost in these strains. We also observed four non-SLR-containing ORFs at the SlrV locus 1: (a) a hypothetical protein with a predicted hydrolase/metallo-beta-lactamase domain (COG2333) which is present in 6 strains (cluster417 in Fig 2); (b) a second hypothetical protein also with a metal-dependent hydrolase/beta-lactamase domain (COG1234) which is only present in the PittHH strain (cluster1434 in Fig 2); (c) a hypothetical protein with a conserved uncharacterized domain (COG3883) and two trans-membrane domains which is present in PittGG and R1838 (cluster754 in Fig 2); and (d) a hypothetical protein only found in strain R1838 (cluster1770 in Fig 2).

Members of the much less prevalent SlrV subfamilies were found at three other genomic loci. In strain PittGG, *slrVE* and *slrVF* are found inserted between a hypothetical *ycbL* homolog and a 2-oxoglutarate dehydrogenase E1 component (SlrV locus 2). In strain R1838 *slrVG* is found inserted between an Undecaprenyl-phosphate N-acetylglucosaminyl 1-phosphate transferase and (Protein-PII) uridylyltransferase (SlrV locus 3). And finally in strain 3655 *slrVH* and *slrVI* are found in tandem between a methionyl-tRNA synthetase and an Apb scaffold protein gene.

### Orthologues with similar motifs

BLAST searches of the SlrV sequences against the non-redundant NCBI database revealed that this family is highly conserved across many bacterial species, including multiple genera and species that colonize the respiratory track such as *Neisseria sp*, *Moraxella catarrhalis*, and *Legionella pneumophila*. SLR-containing genes have been functionally characterized in *L*. *pneumophila* (LpnE, EnhC and LidL) [61–66], *Helicobacter pylori* (Hcp family) [67–72], *Francisella tularensis* (DipA) [73, 74] and *Escherichia coli* (EsiB) [75, 76] where SlrV homologues have all been shown to be critical for host interactions, in particular, intracellular interactions. In all of these species the consensus SLR motif is 36 amino acid residues long and contains the characteristic conserved alanine and glycine residues (Fig 1).

### Distribution of SLR-containing genes among 210 *H*. *influenzae* strains, and correlation with virulence

**Fig 4:**
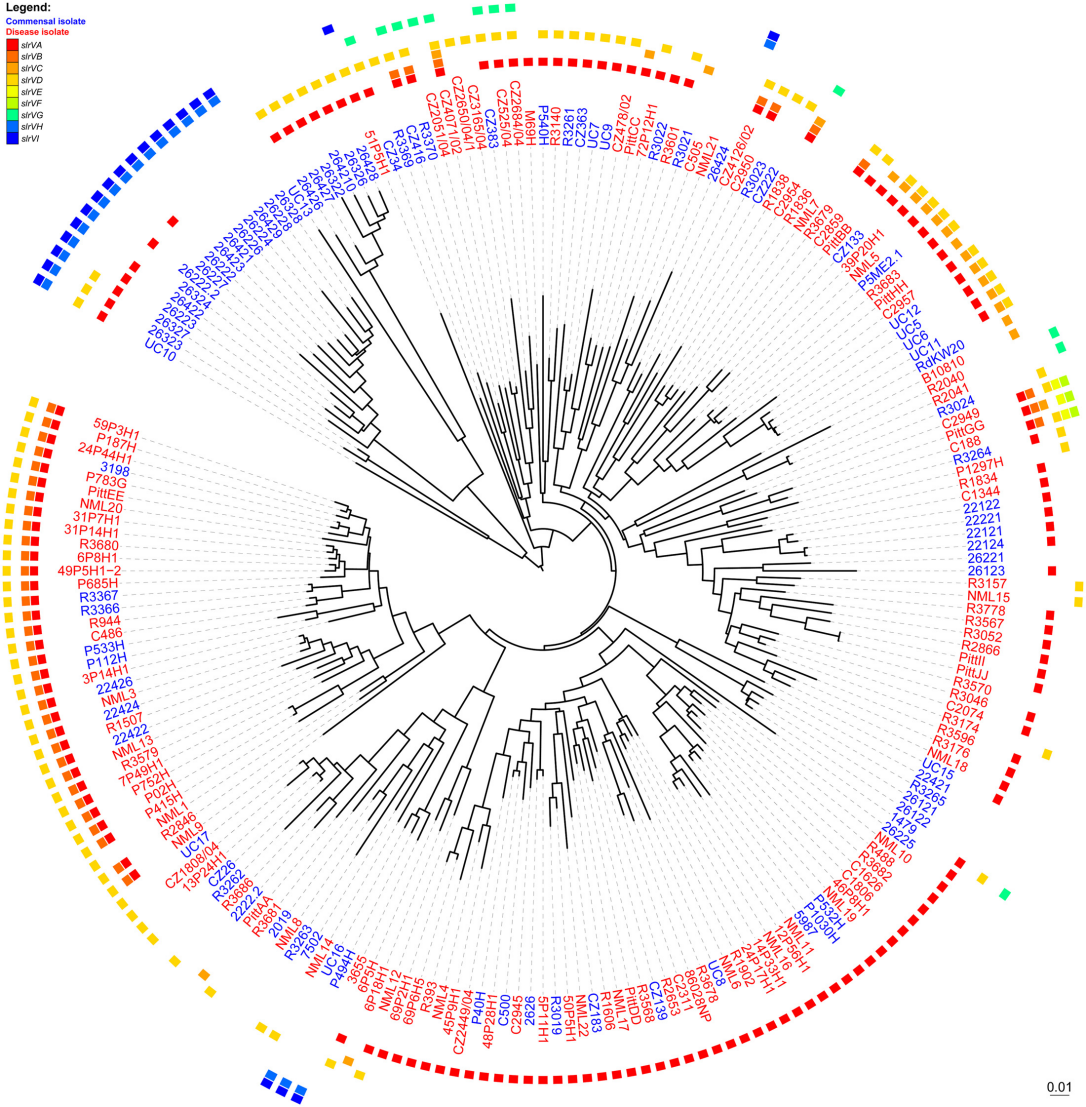
Phylogenetic tree of 210 *H*. *influenzae* strains with the distribution of *slrV* genes and phenotype. Colored blocks indicate the presence of each type of *slrV* gene (*slrVA-I*, see legend). Colored strain names indicate whether the strain is a commensal isolate (blue) or disease isolate (red). Gene data was obtained by whole genome sequencing (24 strains) and by genome hybridization using the custom-designed *H*. *influenzae* SGH array (186 strains) [19]. Binary data (gene presence or absence) was used to build a distance matrix and the phylogenetic tree was calculated using the neighbor joining method [114]. The interactive Tree of Life web server (http://itol.embl.de) was used to visualize the un-rooted tree [112, 113].

Because *slrC* is core to *H*. *influenzae*, no simple association can be made between the presence of SLRs and virulence, however we considered whether the possession of a particular *slrV* or subset of the accessory *slrV* subfamilies might be associated with colonization outside of the nasopharynx. In addition to the 24 sequenced strains, we mined gene possession data for another 186 *H*. *influenzae* strains from a dataset generated from a custom-designed supragenome-based genomic hybridization (SGH) array [19]. This array contains 31,307 probes that collectively cover all known alleles of 2890 of the gene clusters identified from the 24 WGS strains. This includes 299 probes specific to the nine SlrV subfamilies. The *H*. *influenzae* SGH array was used to determine gene presence/absence profiles for each subfamily. However, this method was unable to capture gene copy number or SLR motif copy number. Therefore, statistical associations with copy number were not evaluated below. For the WGS strains, the array analysis accurately identified the presence and correctly assigned the identity of all of the previously detected SlrV, providing confidence that the application of this technology to unsequenced strains would provide robust and accurate data with respect to distribution. The SGH analysis confirmed that *slrC* is a core gene being identified in 209/210 of the strains. A follow-up PCR confirmed that the one *slrC*-negative strain was a false negative (S3 Table). SGH analysis also confirmed that the SlrV family is widespread within the species, such that 92% (193/210) of strains contain at least one member (Fig 4, S3 Table). The most prevalent SlrV subfamily was confirmed to be SlrVA (which we have named *msf*) which was identified in 153/210 strains (Fig 4, *msf* possession is indicated by a red block in the outer track) and is the subject of all of the detailed characterizations reported in this study. SlrVB (43/210, dark orange blocks in Fig 4), SlrVC (21/210, orange blocks in Fig 4) and SlrVD (98/210, light orange blocks in Fig 4) are usually only present in strains that also contain SlrVA. SlrVG is found in 12/210 strains interspersed in the population (green blocks in Fig 4). SlrVE and SlrVF are rare and present in only three closely-related strains (yellow and lime green blocks in Fig 4). SlrVH (20/210) and SlrVI (21/210) are highly correlated and both are present in 21/210 strains; 16 of which are grouped together into a distinct lineage (blue blocks in Fig 4).

We considered the hypothesis that isolates collected from the site of infection of diseased individuals have greater virulence potential than nasopharyngeal carriage isolates taken from healthy individuals due to the presence of accessory virulence factors or a lack of commensal factors [19, 77, 78]. To test whether the *slrV* genes fit this hypothesis, we determined their gene frequencies within the carriage and disease isolate subgroup. Indeed, we found that the fraction of isolates containing either *slrVA* (*msf)* or *slrVB* was significantly higher among disease isolates than carriage isolates (p-values of 0.027 and 0.009 respectively, Fisher-exact test) (Table 3). This analysis also found that *slrVH* and *slrVI* are highly correlated with carriage isolates, as supported by the gene possession tree (Fig 4, Table 3). This difference supports the notion that individual SlrV subfamilies have different biological functions.

**Table 3:** Frequencies of *slrV* genes in various subsets of 210 *H*. *influenzae* isolates.

NTHi strains containing:	All (present/total)	Disease (present/total)	Carriage (present/total)	p-value*
Any *slrV*	193/210 (92%)	116/123 (94%)	77/87 (89%)	0.1978
*slrVA* (*msf*)	153/210 (73%)	97/123 (79%)	56/87 (64%)	**0.0271**
*slrVB*	43/210 (20%)	33/123 (27%)	10/87 (11%)	**0.0087**
*slrVC*	21/210 (10%)	13/123 (11%)	8/87 (9%)	0.8185
*slrVD*	98/210 (47%)	57/123 (46%)	41/87 (47%)	1
*slrVE*	3/210 (1%)	3/123 (2%)	0/87 (0%)	0.2686
*slrVF*	3/210 (1%)	3/123 (2%)	0/87 (0%)	0.2686
*slrVG*	12/210 (6%)	7/123 (6%)	5/87 (6%)	1
*slrVH*	20/210 (10%)	1/123 (1%)	19/87 (22%)	**2.16 x 10**^**−07**^
*slrVI*	21/210 (10%)	2/123 (2%)	19/87 (22%)	**1.49 x 10**^**−06**^

*2-tailed Fisher-exact test comparing gene frequencies in Disease isolates to Carriage isolates

### Selection of PittII as a model strain for *in vitro* and *in vivo* studies

We elected to investigate the SlrVA (*msf*) subfamily due to its prevalence within *H*. *influenzae*, its over-representation among disease isolates, and the fact that *slrVB* is only present when *slrVA* is also present. To characterize the contribution of the SlrVA subfamily in NTHi virulence we selected strain PittII, isolated from a child with perforating otorrhea. This strain was chosen because it: (a) provides a good baseline to observe decreases in virulence, since it induces rapid and severe local and systemic disease in the chinchilla model of OM and invasive disease (OMID) [20]; (b) is more easily transformed than other strains; and (c) codes for only the *slrVA* (*msf)* genes. The SlrV locus 1 in PittII codes for four sequential *msf* genes (Fig 2). These genes have been named *msfA1* (447 base-pairs [bp]), *msfA2* (660 bp), *msfA3* (447 bp), and *msfA4* (660 bp). Although exactly the same size, *msfA1* and *msfA3* share 90% identity, whereas *msfA2* and *msfA4* share only 82% identity. This leads to significant differences in predicted amino acid sequences with 80% identity between *msfA1*/*msfA3* and only 73% between *msfA2*/*msfA4* (S4 Table). Each coding sequence is separated by 187 bp, but no promoters or termination sequences were detected in these regions, suggesting that the whole locus acts as a single operon. To assess the role of the *msf* in NTHi disease, we constructed a knockout (KO) with all four *msf* copies deleted in PittII and replaced with a kanamycin resistance cassette producing the strain PittII Msf-KO (Table 4).

**Table 4:** Strains used in this study.

Strain	Genotype	Antibiotic resistance	Description
PittII	wild-type	-	Clinical OM isolate
PittII GFP	PittII / pRSM2211-GFP	kanamycin	prsm2211 plasmid carrying GFP
PittII Msf-KO	PittII *msfA1-4*::km^R^	kanamycin	Deletion of all four *msf* genes with kanamycin resistance cassette
PittII Msf-COMP	PittII *msfA1-4*::km^R^, *ompP1*::*msfA1*,cm^R^	kanamycin / chloramphenicol	*msfA1* and chloramphenicol cassette inserted *ompP1* locus of PittII Msf-KO
PittII OMPP1-KO	PittII *ompP1*::cm^R^	chloramphenicol	*ompP1* disruption with chloramphenicol resistance cassette
86-028NP	wild-type	-	Clinical NP isolate
86-028NP Msf-KO	86-028NP *msfA1-4*::km^R^	kanamycin	Deletion of all four *msf* genes with kanamycin resistance cassette
RdKW20	wild-type	-	Non-encapsulated variant of a capsular type D strain
RdKW20 Msf-INS	RdKW20 *ompP1*::*msfA1*,cm^R^	chloramphenicol	*msfA1* amplified from strain PittII integrated into *ompP1* locus of strain Rd

GFP: Green fluorescent protein

### PittII *msf* genes are transcribed *in vivo* in the chinchilla OMID model

**Fig 5:**
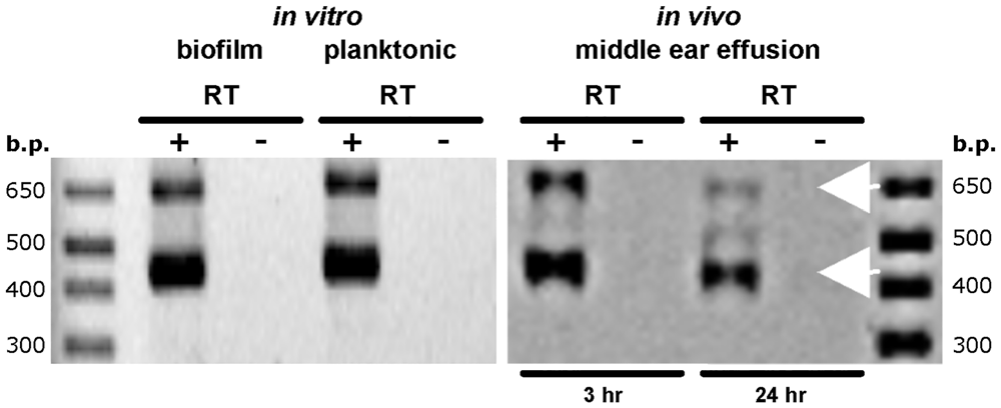
Detection of *msfA* transcripts *in vitro* and *in vivo*. Left panel: RNA was extracted from PittII grown planktonically and as a biofilm in sBHI medium. Right panel: PittII was inoculated bilaterally into the middle ears of three chinchillas via transbullar injection. Animals were euthanized 3h and 24h post-inoculation. Effusions from the middle-ears were harvested immediately and RNA was extracted. Both panels: RNA samples were reverse transcribed (+) or had reverse transcriptase (RT) omitted from the reactions (-). PCR was then performed on + and - RT samples with a primer pair specific to the *msfA* genes. Due to sequence similarity multiple alleles are amplified. The two different sizes of the four *msfA* genes make them easily discernible in the gels (white arrows).

To determine if *msfA1-4* are transcribed under various growth conditions we examined planktonic cultures, *in vitro* biofilms, and bacteria recovered from the tympanic bullae of PittII-infected chinchillas. We detected transcripts under all three conditions, consistent with the predicted 447 and 660 bp ORFs, confirming that at least two separate *msf* genes were transcribed (Fig 5).

### Msf is important in *in vitro* macrophage uptake and survival

**Fig 6:**
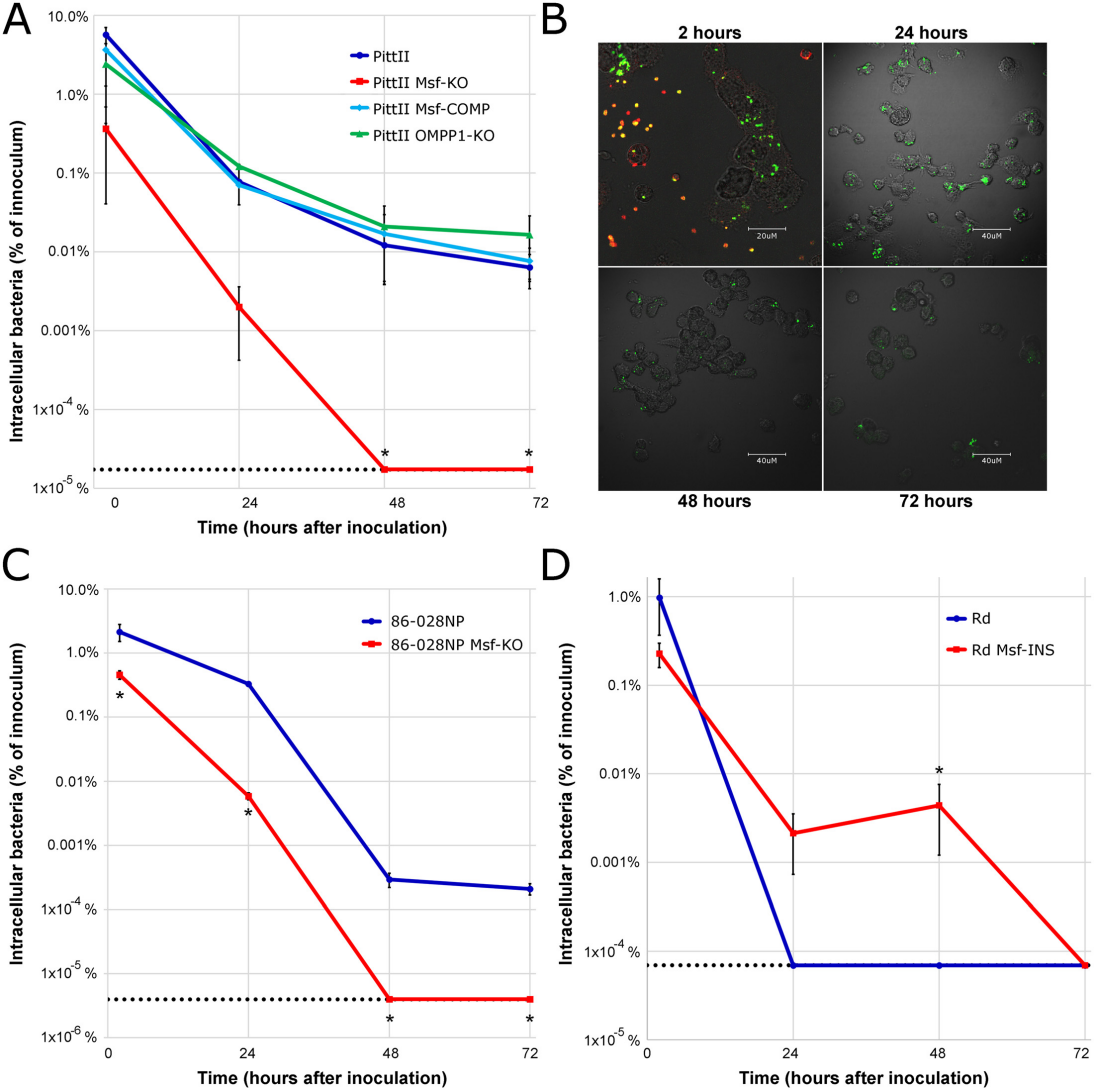
The PittII Msf-KO strain has decreased survival in macrophages. Polymyxin B protection assays showing the number of viable bacteria recovered from THP-1 macrophage monolayers 2, 24, 48 and 72 hours after inoculation. Each result represents the mean of 3 wells in 3 biological replicate experiments. **(A)** Bacterial uptake and survival of: PittII (clinical OM isolate), PittII Msf-KO, the single *msfA1* gene inserted into the Msf-KO at the *ompP1* locus: PittII Msf-COMP, and a PittII OMPP1-KO control. Dotted line indicates the limit of detection for the Msf-KO strain. * p<0.05 by one-way weighted ANOVA for independent samples and p<0.05 by Tukey HSD post-hoc test for Msf-KO compared to WT, Msf-COMP and OMPP1-KO. **(B)** Differentiated THP-1 macrophage monolayers were infected with PittII expressing GFP. At each time-point the cells were washed and fixed. Samples were stained using rabbit anti-NTHi and Alexa Fluor secondary antibodies (red). Red staining indicates extracellular bacteria that are dead. Yellow/orange indicate extracellular bacteria that are viable (expressing GFP). Intracellular bacteria appear green since macrophages were not permeabilized. After 24 hours differential staining of extracellular and intracellular bacteria shows that the majority of the bacteria are inside the macrophages. **(C)** Bacterial uptake and survival of: NTHi strain 86-028NP (clinical OM isolate) and 86–028 Msf-KO. Dotted line indicates the limit of detection for the Msf-KO strain. * p<0.05 by two-tailed *t*-test for two independent means. **(D)** Bacterial uptake and survival of: Rd KW20 (non-encapsulated variant of a type D strain that lacks any Msf gene) and Rd Msf-INS (A mutant with the PittII *msfA1* gene inserted at the *ompP1* locus). Dotted line indicates the limit of detection for the Rd KW20 strain. * p<0.05 by two-tailed *t*-test for two independent means.

The *L*. *pneumophilia* and *F*. *tularensis* Msf homologues (LpnE, EnhC, and DipA respectively) all play roles in macrophage survival [62–65, 73]. Furthermore, it has been proposed that an ability to survive within human host cells contributes to *H*. *influenzae* persistence and/or trafficking to new infection sites [36–49]. Thus, we compared the ability of WT and KO PittII strains to invade and survive in human macrophages to determine the role of Msf in phagocytosis and intracellular persistence. We inoculated differentiated THP-1 macrophages at a MOI at 100:1 and incubated for 1h to allow for adherence and phagocytosis. Extracellular bacteria were then eliminated with polymyxin B, the macrophages were lysed, and the viable intracellular bacteria were enumerated using dilution and plate counts at 2, 24, 48 and 72 hours after inoculation (Fig 6A).

At the 2 hour time point, there were ~15X more WT than KO bacteria within the macrophages. Furthermore, the WT was able to survive up to 72 hours, whereas the mutant strain was completely killed within 48 hours (Fig 6A). The survival defect of the KO was rescued by complementation with a single *msf* gene, *msfA1*, demonstrating that a single allele is sufficient for extended survival within macrophages (strain PittII Msf-COMP). The complemented strain was created by inserting *msfA1* into the *ompP1* site, but PittII survival in macrophages was not affected by the deletion of the *ompP1* gene as demonstrated with control strain PittII OMPP1-KO (Table 4). We confirmed that the bacteria were intracellular by using a combination of a fluorescent reporter strain and inside-out staining (Fig 6B).

The effect of Msf loss on macrophage survival is not strain-specific, since the presence of SlrVA subfamily is also critical for macrophage survival of strain 86-028NP. Like PittII, the 86-028NP strain contains four adjacent *msf* genes (Fig 2). In this strain, a deletion mutant of the entire SlrV locus 1 (86-028NP Msf-KO) is unable to survive 48 hours after invasion, while the WT survives more than 72 hours (Fig 6C).

Furthermore, the Rd KW20 strain, which lacks any *slrV* gene (Fig 2), has been previously shown to be quickly phagocytosed and killed within 24 hours by macrophages [50]. Yet, when *msfA1* is inserted into Rd KW20 (Rd Msf-INS), it survives substantially and significantly better than WT, lasting up to 48 hours within macrophages (Fig 6D).

Phagocytosis by human macrophages requires rearrangement of the actin cytoskeleton, which is inhibited by cytochalasin D. In the absence of this inhibitor, after one hour 16.46±0.12% of the PittII were found within the PMA-differentiated THP-1 cells, while in the presence of cytochalasin D the vast majority of the PittII cells remained in the extracellular compartment (invasion rate of 1.48±0.11%). This effect suggests that PittII primarily enters macrophages via an actin-dependent process.

### Msf confers PittII with an *in vivo* fitness advantage

To determine whether Msf provides a fitness advantage, we competed WT and Msf-KO PittII strains in: 1) planktonic culture; 2) *in vitro* biofilms; and 3) *in vivo* using the chinchilla OMID model. In these assays, the WT and kanamycin resistant mutant strains were mixed in a 1:1 ratio and then inoculated bilaterally through the tympanic bullae and allowed to infect for three days. In each assay, end point samples were serially diluted and plated on two sets of agar plates: non-antibiotic-containing plates to enumerate the total amount of bacteria present and kanamycin-containing plates to enumerate the KO only. The competitive Index (CI) was calculated as the ratio of the colony-forming units (CFU) of KO to WT recovered, adjusted for initial input (KO end / WT end) / (KO_t = 0_ / WT_t = 0_).

**Fig 7:**
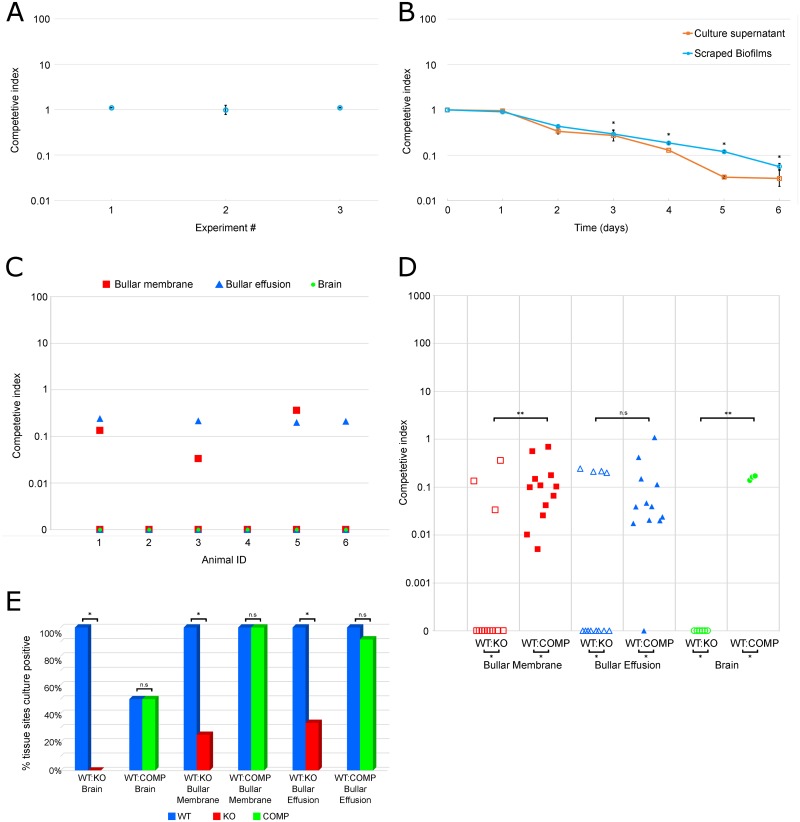
PittII Msf-KO strain suffers a large competitive disadvantage compared to WT. Competitive Index (CI) refers to a ratio of mutant bacteria to WT bacteria adjusted for the initial inoculum ratio. CI of 1 indicates no competitive advantage. Data points below 1 indicate a WT advantage and data points above 1 indicate a mutant advantage **(A-D)**. **(A)** PittII WT and Msf-KO inoculated 1:1 in planktonic culture for 24 hours (3 experiments with n = 3, error bars denote the standard deviation of each experiment). **(B)** PittII WT and Msf-KO inoculated 1:1 in biofilm culture harvested over a 6 day period (3 experiments with n = 3, error bars denote the standard error of the mean). CFU were enumerated from both the adherent biofilms as well as the overlying supernatant and the CI was determined from each fraction. *p<0.05 by one-sample two-tailed *t*-test (μ_0_ = 0) on the log CI for both the biofilm and planktonic fractions. **(C-E)** In two separate experiments six chinchillas were inoculated with 1:1 mixtures of strains and five tissue sites (brain, right and left bullar membranes and right and left bullar effusions) were harvested three days after inoculation. Thus n = 6 for brains and n = 12 for bullar sites. **(C)** *In vivo* competition between PittII WT and Msf-KO inoculated 1:1 into 6 animals. Each data point represents a single tissue-site CI value. Points on the X-axis (CI of 0) indicate that no KO bacteria were observed and therefore have an infinitely low CI value. **(D)** *In vivo* competition between PittII WT and Msf-COMP inoculated 1:1 into 6 animals compared with the previous data obtained from competition between PittII WT and Msf-KO *in vivo*. Each data point represents a single tissue-site CI value. Points on the X-axis (CI of 0) indicate that no KO bacteria were observed and thus have an infinitely low CI value. *p<0.05 by two-tailed *t*-test for two independent means of log cfu data. **p<0.05 by Mann-Whitney U test. n.s. (not significant). **(E)** Percentage of tissue sites that were positive for bacteria in each of the two *in vivo* competition experiments (WT vs Msf-KO, and WT vs Msf-COMP). * p<0.05 by two-tailed Fisher-exact test. n.s. (not significant).

In planktonic cultures we detected a slight difference in growth rate between the PittII WT and Msf-KO strain, however both grew to the same maximum OD_A600_ (S2 Fig). Despite this there was no significant difference in the fitness of the WT and KO strains during co-culture: for 3 independent experiments, CI = ~1 (Fig 7A). In contrast, when bacteria were grown as biofilms, the WT strain displayed a strong advantage starting on day 2 and dominated the cultures by day 4 (Fig 7B) as indicated by decreasing CI. The WT’s advantage was observed both in the biofilm itself, as well as in the supernatant where detached bacteria are found and was statistically significant from Day 3 onwards (p<0.05, one-sample two-tailed *t*-test). This suggests that the difference does not reflect variability in attachment between the strains.

Equal numbers of WT and KO were used to bilaterally infect the middle ears of six chinchillas. After 3 days, the animals were euthanized and the bacteria were collected from: left and right ear effusions; the adherent biofilm layers attached to the middle-ear mucosa (left and right bullar membranes); and the brains. In all 6 animals and all 5 collections, the WT strain displaced the KO strain almost completely (Fig 7C).

The single *msfA1* complement strain (PittII Msf-COMP) did not fully restore the WT phenotype during *in vivo* infections, since they were still outcompeted by the WT strain, albeit to a lesser degree (Fig 7D). This is in contrast to the macrophage phagocytosis/survival experiments where the phenotype was fully complemented (indicating that the slight growth defect is irrelevant in these assays) (Fig 6A). As shown above, in the WT:KO competition experiment, very few tissue sites had any detectable KO bacteria after three days (3/12 bullar membranes and 4/12 bullar effusions). In contrast, in the WT:COMP competition, both the WT and the Msf-COMP strains were recovered from almost all tissue-sites (Fig 7E). Of particular note the MsfA1 complementation restored trafficking to the brain, as we detected both WT and Msf-COMP bacteria in 3/6 brains.

### Msf plays a role in NTHi dissemination to the brain in the chinchilla OMID model

**Fig 8:**
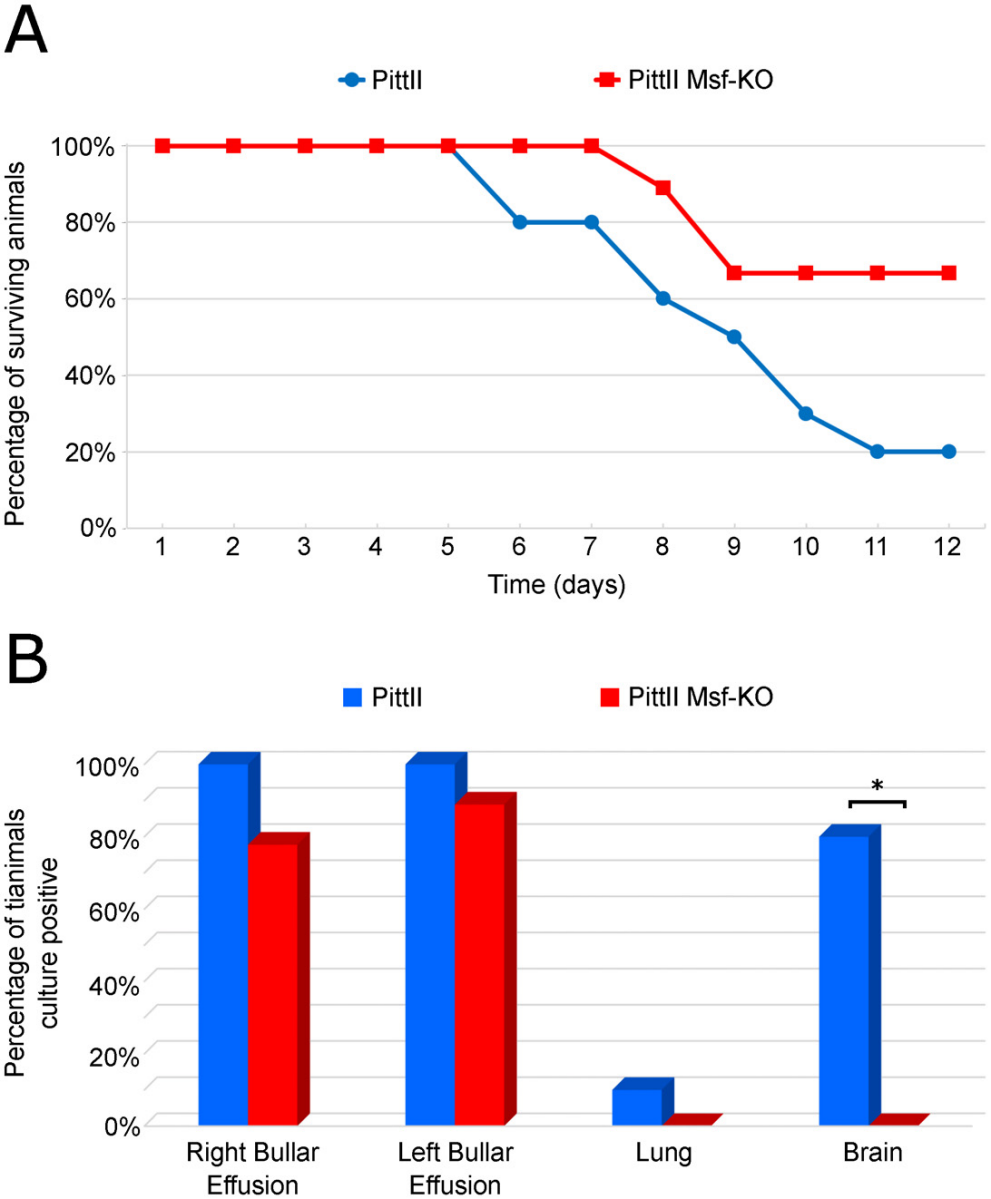
Differences in invasiveness and mortality between the PittII WT and Msf-KO strains in the chinchilla OMID model. Two cohorts of chinchillas were inoculated bilaterally through the tympanic bulla with either PittII or PittII Msf-KO. (A) Mortality over time showing the number of animals still alive after each day; (B) Bacterial recovery percentages by tissue for WT and Msf-KO infected animals. Tissues were collected at the time of animal death. * p<0.05 by two-tailed Fisher-exact test.

WT and Msf-KO strains were separately evaluated to ascertain the virulence effect of the *msf* on NTHi virulence and disease progression. Strains were inoculated bilaterally through the tympanic bullae and animals were monitored daily for up to 12 days for signs and severity of local (otologic) and systemic disease (See S5 Table for scoring criteria). All animals developed bilateral OM, though we detected no significant difference between the WT and Msf-KO strains with respect to local middle ear disease (data not shown). However, the mortality between the two groups was noticeably different (although not statistically significant due to the small number of animals infected). Only two out of the ten WT infected animals survived until the end of the experiment, whereas six of nine animals infected with the KO strain survived (p = 0.0698, Fisher-exact test) (Fig 8A). In addition to observing clinical signs during disease progression, upon death the left and right bullar effusions, the brain, and the lungs were collected and analyzed for the presence of WT and Msf-KO bacteria (Fig 8B). Consistent with the *in vivo* competition experiment, the WT strain was recovered from the brain in 8 out of 10 animals, while the Msf-KO strain was not detected in the brain of any animals (0/10) (p-value = 0.0007, Fisher-exact test). This difference along with the competition data suggests an important role for *msf* in dissemination to the CNS and/or blood in this model.

### Msf is important in anaerobiosis

We compared the transcriptional profiles of the PittII WT and Msf-KO strains during late exponential/early stationary phase planktonic culture (OD_A600_ of 0.7) using the *H*. *influenzae* SGH Array and the methods outlined by Janto *et al* [79]. The threshold for differentially regulated genes was set as an absolute change of at least 2-fold and with a Bonferroni-corrected p-value of 0.05 or less. The Msf-KO strain had 75 up-regulated and 75 down-regulated genes compared to the WT (S6 Table). Raw and processed transcriptional data for this experiment has been deposited in NCBI's Gene Expression Omnibus (GEO) [80] and are accessible through GEO Series accession number GSE70172 (http://www.ncbi.nlm.nih.gov/geo/query/acc.cgi?acc=GSE70172).

Many genes involved in anaerobic respiration were down-regulated in the KO strain (Table 5), including: *arcA*, part of the two component system ArcAB; an oxygen-sensitive master regulator, and the *nrfABCD* operon which encodes a periplasmic nitrite reductase. Under anaerobic conditions, the gene products from the *nrf* operon reduce nitrate to ammonia in the bacterial periplasm [81, 82]. In addition, the Nrf complex has been implicated in virulence through the detoxification of nitric oxide (NO), which is important in macrophage survival [81, 83].

**Table 5:** Genes differentially regulated in the PittII Msf-KO strain compared to the WT strain that are associated with virulence or anaerobiosis.

SGH-Array Cluster ID	WT-avg	KO-avg	FOLD	Bon pVal	ANNOTATION
cluster2470b	**13281**	**318**	**-41.71**	<1E-16	Tripeptide aminopeptidase (EC 3.4.11.4)
cluster2051k	**1247**	**49**	**-25.47**	2.08E-12	SlrVA protein (macrophage survival factor, msfA), Sel1-like repeat
cluster2554	**3054**	**160**	**-19.11**	5.08E-09	Antiholin-like protein LrgA
cluster2197	**15370**	**1139**	**-13.49**	2.08E-13	lrgA-associated membrane protein LrgB
cluster3031	**23039**	**1161**	**-19.84**	<1E-16	Putative oxidoreductase component of anaerobic de-hydrogenases; Chaperone protein TorD
cluster231a	**35163**	**2771**	**-12.69**	<1E-16	DmsC, Anaerobic dimethyl sulfoxide reductase chain C(EC 1.8.99.-)
cluster9	**5634**	**453**	**-12.44**	2.08E-12	Ferredoxin-type protein NapF (periplasmic nitrate reductase)
cluster2028	**39430**	**3932**	**-10.03**	<1E-16	DmsB, Anaerobic dimethyl sulfoxide reductase chain B(EC 1.8.99.-)
cluster2113	**34130**	**4302**	**-7.93**	<1E-16	DmsA, anaerobic dimethyl sulfoxide reductase chain A
cluster2142	**21050**	**2728**	**-7.72**	5.27E-11	Cytochrome c-type protein NrfB
cluster947	**22732**	**3112**	**-7.30**	6.36E-10	NrfC protein
cluster1427	**23876**	**4006**	**-5.96**	1.32E-09	NrfD formate-dependent nitrite reductase membrane component
cluster2328	**42169**	**9033**	**-4.67**	2.84E-11	Cytochrome c552 precursor (EC 1.7.2.2) NrfA
cluster599	**3329**	**518**	**-6.43**	1.73E-03	Molybdenum cofactor biosynthesis protein A, MoaA
cluster320	**4404**	**1282**	**-3.44**	1.80E-06	Molybdenum cofactor biosynthesis protein D; Molybdopterin converting factor subunit 1, MoaD
cluster47	**7095**	**2493**	**-2.85**	8.78E-06	Molybdenum cofactor biosynthesis protein C, MoaC
cluster581	**11177**	**2113**	**-5.29**	2.54E-09	ABC transporter involved in cytochrome c biogenesis ATPase component CcmA
cluster6	**8347**	**1664**	**-5.02**	6.83E-09	ABC transporter involved in cytochrome c biogenesis CcmB
cluster230	**10899**	**2220**	**-4.91**	9.50E-07	Cytochrome c-type biogenesis protein CcmD interacts with CcmCE
cluster544	**7343**	**1624**	**-4.52**	1.98E-07	Cytochrome c-type biogenesis protein CcmC putative heme lyase for CcmE
cluster1241	**9123**	**2686**	**-3.40**	3.41E-07	Cytochrome c heme lyase subunit CcmF
cluster1025	**2451**	**863**	**-2.84**	3.82E-03	Cytochrome c-type biogenesis protein CcmG/DsbE thiol:disulfide oxidoreductase
cluster419	**22511**	**8061**	**-2.79**	1.88E-05	Cytochrome c-type biogenesis protein CcmE heme chaperone
cluster735	**6383**	**2438**	**-2.62**	6.88E-05	Cytochrome c heme lyase subunit CcmL
cluster2103	**6210**	**1306**	**-4.75**	1.64E-06	Nitric oxide-dependent regulator DnrN or NorA
cluster2947b	**37123**	**9336**	**-3.98**	1.91E-10	TorY, Cytochrome c-type protein
cluster3059	**41761**	**15459**	**-2.70**	6.45E-08	TorZ, Trimethylamine-N-oxide reductase (TMAO) (EC 1.6.6.9)
cluster3019	**49753**	**20427**	**-2.44**	2.59E-07	C4-dicarboxylate like transporter
cluster1162	**25682**	**12292**	**-2.09**	2.30E-03	Aerobic respiration control protein ArcA
cluster478	**1553**	**26795**	**17.25**	1.95E-11	Candidate type III effector Hop protein
cluster2202	**7564**	**34197**	**4.52**	1.60E-05	ATP-dependent hsl protease ATP-binding subunit HslU
cluster1261	**2285**	**8453**	**3.70**	5.63E-05	Manganese superoxide dismutase (EC 1.15.1.1) SodA

WT-avg and KO-avg: Average expression values for PittII WT and Msf-KO strains. FOLD defined as KO expression level relative to WT. Bon pVal: Bonferroni-corrected p-value. Genes in likely operons or found in tandem on the chromosome are grouped together and ordered roughly by the magnitude of fold changes.

The *ccmABCDEFGHL* operon, of which all components were found down-regulated in the KO, encodes a type 1 cytochrome *c* biogenesis system including a heme exporter that is required for cytochrome *c* maturation [84, 85]. Thus, the Ccm complex plays an essential role in electron transfer and respiration.

Also down-regulated in the KO strain were the *dmsABC/torD/napF* and *torYZ* operons. The Dms complex encodes a dimethlyl-sulfoxide (DMSO) reductase, and *torD* and *napF* encode components of the trimethylamine N-oxide (TMAO) and nitrate reducing complexes, respectively. DMSO reductase is a membrane-associated anaerobic electron transfer enzyme that contains molybdenum and iron-sulfur cofactors [86, 87]. In addition to DMSO this complex can act on various other methyl-sulfoxides including TMAO and is transcriptionally activated in the absence of nitrate and oxygen [86–88]. The Tor gene products make up a TMAO reductase that is functionally related to the Dms DMSO reductase due to overlapping substrates [89]. Finding both Dms and Tor operons cumulatively strengthens the theme of anaerobic and nitrogen regulated genes involved in electron transfer. Like Nrf and Dms, Tor gene products require molybdenum cofactors [90]. Keeping in this theme, the *moaA-D* operon was also found down-regulated in the KO strain, which encodes molybdenum cofactor biosynthesis genes.

Finally, in addition to anaerobiosis genes, several virulence factors were found differentially regulated between the WT and KO strain. Highly down-regulated in the KO strain were the LrgAB virulence factors which encode an anti-holin like complex that increases penicillin tolerance and inhibits murein hydrolase channels in *Staphylococcus aureus* [91]. Highly up-regulated in the KO strain was a Hop effector protein associated with Type III secretion as well as manganese superoxide dismutase (*sodA*).

Ten of these genes (each from a different operon) were chosen for confirmation by quantitative real-time PCR (qRT-PCR). Primers were designed for each of these genes based on the PittII WGS data (S7 Table). The microarray results were confirmed in all cases by the qRT-PCR (Table 6).

**Table 6:** qRT-PCR confirmation of microarray-based transcriptomic results.

SGH Array cluster ID	Gene	Annotation	Microarray fold change*	qRT-PCR fold change*
cluster2470b	-	Tripeptide aminopeptidase (EC 3.4.11.4)	-41.71	-93.72
cluster2197	*lrgB*	lrgA-associated membrane protein LrgB	-13.49	-20.87
cluster9	*napF*	Ferredoxin-type protein (periplasmic nitrate reductase)	-12.44	-19.70
cluster2142	*nrfB*	Cytochrome c-type protein	-7.72	-14.39
cluster232	*nrfA*	Cytochrome c552 precursor (EC 1.7.2.2)	-4.67	-13.05
cluster581	*ccmA*	ABC transporter involved in cytochrome c biogenesis ATPase component	-5.29	-6.57
cluster599	*moaA*	Molybdenum cofactor biosynthesis protein A	-6.43	-3.04
cluster1261	*sodA*	Manganese superoxide dismutase (EC 1.15.1.1)	3.70	4.90
cluster2202	*hslU*	ATP-dependent hsl protease ATP-binding subunit	4.52	17.61
cluster478	-	Candidate type III effector Hop protein	17.25	37.82

*fold change defined as KO relative to WT

## Discussion

We have reported and characterized a large and heterogeneous set of genes in *H*. *influenzae* that contain Sel1-like repeats (SLR). The SLR acronym is derived from the first characterized member, the *C*. *elegans* suppressor-enhancer of lin-12 (sel) gene, but refers specifically to the motif found among bacteria (Pfam #PF08238) [92]. Proteins with SLR domains are a subgroup within the solenoid protein superfamily, which includes tetratricopeptide repeat (TPR, 34 aa repeats) proteins, pentatricopeptide repeat (PPR, 35 aa repeats) proteins and transcription activator-like (TAL) effectors (30–42 aa repeats). Tandem arrays of amino acid repeats in these proteins lead to the formation of modular secondary structures such as sets of anti-parallel α-helices and result in a superhelical macromolecule. Functionally, some TPRs have been implicated in protein-protein interactions [93], some PPRs show RNA-binding capability [94] and some TAL effectors have been demonstrated and exploited to bind DNA [95, 96]. SLRs contain 36–44 aa repeats and are characterized by conserved glycine residues that support sharp turns in the superhelices as well as conserved alanine residues [76, 97]. Consistent with this, the 36-residue SLR motif we found in *H*. *influenzae* contains four highly conserved glycine and alanine residues (positions 4, 11, 12, 15, 19, 24, 30, and 32: Fig 1).

The highly conserved residues allow for identification of the motif, yet there is considerable heterogeneity associated with SlrV genes, which occurs at multiple levels including: the presence of at least ten SLR-containing gene subfamilies based on sequence homology; variation in the number of motif repeats with a gene subfamily; variation in the number of gene copies per strain; variation in the number of different gene subfamilies per strain, and variation with respect to chromosomal location based on gene subfamily type (Figs 1–4). The modularity of SLR-containing genes allows for rearrangement of the modular units, as well as expansion and contraction of tandemly repeated SLR domains. In this manner, SLR-containing genes have the potential to rapidly evolve. We hypothesize multiple adaptive values for the changes. First, they could affect protein function by changing the binding properties of the SLR-containing protein and its partners. Alternatively, they could misdirect the immune response by focusing it on decoy peptide that is highly variable yet functionally irrelevant [98].

One SLR subfamily, SlrC, is found in all strains and highly conserved, whereas the remaining SlrV subfamilies have variable distributions. Although *slrV* genes are found in the majority of *H*. *influenzae* strains, different SLR subfamilies have distinct distributions suggesting that they have distinct functions. Two subfamilies (SlrVH and SlrVI) are associated with commensal isolates. Others (SlrVA and B) are associated with disease isolates (Table 3). We focused our initial studies on SlrVA, since it is the most common SlrV subfamily and is the sole SlrV locus in some strains, allowing us to investigate its function in isolation of the others. This is the case in NTHi strain PittII, a highly virulent strain in the chinchilla OMID model originally isolated from a child with perforating otorrhea, which we used to characterize the SlrVA subfamily (of which *msf* is a member).

In investigating the function of the *slrVA* (*msf*) genes in PittII, we considered functional studies of SLR-containing proteins in other species of bacteria, many of which interact with host proteins. At least nine genes with SLR domains have been identified in *H*. *pylori*; also known as the Helicobacter cysteine-rich protein (Hcp) family, due to the presence of conserved pairs of cysteine residues within each SLR repeat. These cysteines are separated by seven residues and preceded by alanine, glycine or serine [71]. Phylogenetic analyses in *H*. *pylori* found strong positive selection of residues on the SLR surface of Hcps in a gene and lineage specific manner (which for this species is also correlated with geographic location) [68]. These observations suggest that the mutations are adaptations to host responses. In *H*. *influenzae* we found a highly conserved pair of cysteine residues matching the *H*. *pylori* motif, not in the SLR themselves, but as a part of a conserved SlrV C-terminus (Fig 1D). This raises the possibility that the *H*. *influenzae* SlrV proteins also cross-link via di-sulfide bridges similar to the Hcps [71].

Many previously characterized SLR-containing genes in other species are involved in host-pathogen or host-symbiont protein-protein interactions. Hcps are recognized by the host’s immune system, as indicated by anti-Hcp antibodies in sera from *H*. *pylori* patients [99]. HcpC has been shown to interact with the host proteins Nek9, Hsp90 and Hsc71 [70]. HcpA is a potent pro-inflammatory and Th1-promoting protein, and can trigger the differentiation of human myeloid monocytes into macrophages [67, 69]. Six SLR-containing genes have been identified in *L*. *pneumophilia*; three of them (*lpnE*, *enhC* and *lidL*) have been implicated in host interactions, specifically cell entry and/or trafficking of the *L*. *pneumophilia* containing vacuole [61–66]. Consistent with direct host interaction, LpnE is found in culture supernatants [63], is required for invasion of human epithelial and macrophage cell lines [62, 63], is localized to the legionella containing vacuole membrane [65], and can interact with the human proteins OBSL1 [63] as well as OCRL1 and the glycolipid PtdInd(3)P [65]. The intracellular pathogen *F*. *tularensis* also produces an SLR-containing protein, DipA, which has been shown to be membrane-associated and localized to the bacterial surface. Deletion of *dipA*, results in a defect in intracellular replication and survival in macrophages as well as dissemination and lethality in mice [73].

Here we report similar findings in *H*. *influenzae*. Deletion of all four SlrVA genes from *H*. *influenzae* strain PittII revealed a defect in survival within macrophages (Fig 6A). We therefore renamed these genes macrophage survival factors (*msf*). We also observed this survival defect in OM strain 86-028NP upon deletion of its four *msf* genes (whose copies have slightly different numbers of motif repeats: Fig 2). Furthermore, insertion of a single copy of the PittII *msfA1* gene into the avirulent strain Rd KW20 led to an increase in its survival time within macrophages (Fig 6D). All of these data support the hypothesis that *msf* (and therefore the SlrVA subfamily) plays a role in intracellular survival. Competition studies in the chinchilla OMID model showed that the presence of *msf* provided a significant fitness advantage *in vivo*, and vastly increased trafficking to the brain (Figs 7 and 8). Together these two traits probably account for much of the difference in mortality seen between the PittII WT and Msf-KO strains (Fig 8A). Notably, complementation of the PittII Msf-KO mutant with a single *msfA1* gene restored the macrophage survival defect (Fig 6A), but only partially complemented the mutant defect in causing systematic disease in chinchilla (Fig 7D and 7E). This suggests a gene dosage effect *in vivo* that is not observed in macrophage survival *in vitro*. Alternatively, there may be slightly different functions for the various *msf* alleles.

Protein-protein interactions can influence signaling events and there is some evidence for involvement of SLR-containing proteins in signal transduction. The alpha-proteobacterium *Sinorhizobium meliloti* utilizes the two-component system (TCS) ExoS/Chv1 to regulate the switch from its free living to invasive form within its alfalfa host (*Medicago sativa*) by modulating biofilm formation and lipopolysaccharide modification [100–103]. ExoR, which is an SLR-containing protein, represses ExoS/Chv1 signaling by direct binding to ExoS [103]. In this context, it is notable that the *H*. *influenzae* SLRs share a 100% conserved tyrosine residue, which is not common to SLRs in general. We therefore hypothesize that this residue is important for the *H*. *influenzae* specific functions of its SLR-containing proteins. Future work will focus on establishing whether this residue is a kinase target involved in bacterial signaling.

We investigated a role for *msf* in bacterial signaling by performing a microarray analysis. We observed down-regulation of multiple operons that encode periplasmic proteins for utilizing alternative electron acceptors such as nitrate (*nap*), nitrite (*nrf*), and methyl sulfoxides (*dms*, *tor*), as well as genes associated with required cofactors (*moa*). While these operons are under the control of oxygen-sensitive master regulators like Fnr and the TCS ArcAB, we note a much more significant overlap with the regulon that is controlled by the nitrate- and nitrite-sensitive TCS NarPQ [104–107]. Because no transcriptional changes were observed in *narPQ* and due to the propensity of SLR-containing molecules to be involved in protein-protein interactions, we hypothesize that Msf plays a role in NarPQ signaling at the protein level. Alternatively, Msf might be a host-interacting protein that affects the NarPQ regulon indirectly via an unidentified intermediate. Regardless, the transcriptomic differences observed between the WT and KO PittII strains suggest that Msf proteins play an important role in the regulation of genes during under anaerobic conditions (Table 5). *H*. *influenzae* forms robust biofilms during chronic infections [3, 4, 8], and it is known that dissolved oxygen levels drop precipitously within biofilms [108]. Thus, the fitness advantage of the WT over the KO in the *in vitro* biofilm competition assays and the *in vivo* competition assays may exist, in part, because of the WT's ability to sense and respond to a lack of O_2_ as a terminal electron acceptor. The same pathway may also be involved in the macrophage survival phenotype due to oxygen limitation in an intracellular environment. Additionally, it is known that NrfA (which is down-regulated in the Msf*-*KO) consumes NO, thereby minimizing the formation of reactive oxygen species by macrophages [81, 82]. We hypothesize that the importance of Msf in intracellular macrophage persistence explains the reduced invasiveness and inability of the Msf-KO to infect the chinchilla brain *in vivo*. Future work will focus on determining whether this is by direct trafficking within macrophages or whether the intracellular persistence phenotype is relevant to other cell-types as well.

Our data demonstrate that the SLR-containing Msf proteins are virulence factors in *H*. *influenza*e infections, where they likely play a role in both chronicity of disease by providing a fitness advantage in biofilms and increased survival in macrophages, as well as in invasive disease as shown by increased trafficking to the brain in the chinchilla disease model. We propose that other SlrV family members are also likely to be involved in the virulence potential of *H*. *influenzae*. Chronic *H*. *influenzae* infections are usually polyclonal, and on average, strains differ by approximately 20% of their genic content. Further, many strains are not virulent, and eliminating all *H*. *influenza*e strains may lead to adverse changes in the host’s microbiome. The SlrVA (Msf) represent a potential target to eliminate large subsets of highly virulent strains, while allowing strains with less pathogenic potential to remain intact, thus setting the stage for a microbiome-friendly treatment strategy.

## Materials and Methods

### Ethics Statement

All animal work was conducted with the approval of the Allegheny-Singer Research Institute's Institutional Animal Care and Use Committee (IACUC) and Research Facilities Department (RFD). Working closely with the IACUC, the RFD provides the highest standards of humane care and use of laboratory animals and assures compliance with institutional and federal regulations. They share responsibility to assure that the use of animals in research projects are necessary, that the investigator has included in the protocol measures to eliminate any unnecessary pain and discomfort to the animals, and that alternatives to the use of live animals have been considered.

#### *In silico* analysis for domain identification

47,997 coding sequences (CDS) identified in 24 strains of *H*. *influenzae* were interrogated using the Multiple EM for Motif Elicitation (MEME) program [58, 59] (http://meme.nbcr.net/meme/tools/meme). This program is designed to discover domains conserved among sequences by creating a position-dependent probability matrix. Once the 36 amino acid SLR motif had been identified, the consensus sequence from MEME was submitted to the Motif Alignment and Search Tool (MAST) program [60] to search for new instances and variants of the initially identified SLR-containing ORFs. Multiple iterations of MEME/MAST were performed to maximize identification of SLR-containing proteins. Sequence identities and similarities were determined using the BLAST programs and the GenBank non-redundant database on the NCBI web server. Motifs were drawn in R with the help of the motifStack package (http://www.bioconductor.org/packages/release/bioc/html/motifStack.html) (Fig 1 and S1 Fig). Amino acid colors are a modification of the WebLogo default, with Tyr and Cys having unique colors (Y = orange and C = turquoise).

#### Phylogenetic Tree based on SLR gene sequences and domains

We generated multiple sequence alignments (MSA) using Clustal Omega [109] for 1) 79 SLR-containing full-length CDS and; 2) the signal peptide sequences identified from these CDS using SignalP 4.1 n = 78; one sequence was located on a contig break and missing the N-terminus) [110]. Output from MEME/MAST analysis was used as an MSA for the 36 amino acid (aa) SLR motifs as well as a 22 aa motif identified in the C-terminus of 54/55 SlrV CDS. Maximum likelihood trees were built with the MSA as input to the RAxML 8.1.2 software using rapid bootstrapping with convergence test, thorough maximum likelihood search, Gamma distribution, and the WAG aa substitution matrix [111]. The interactive Tree of Life web server (http://itol.embl.de) was used for visualization and to generate Fig 3 [112, 113].

#### Phylogenetic Tree based on gene possession

The ‘ape’ package in the ‘R’ environment was used to 1) build a distance matrix based on the SGH gene presence/absence data using the binary setting and 2) generate a phylogenetic tree based on this distance matrix using the neighbor joining method [114]. The interactive Tree of Life web server (http://itol.embl.de) was used for visualization and to generate Fig 4 [112, 113].

#### PCR and Sanger Sequencing of SlrV locus 1 in *H*. *influenzae*

Genomic DNA extractions were performed on 210 *H*. *influenzae* isolates using the QIAamp DNA Mini Kit (Qiagen, CA) according to the manufacturer’s instructions for Gram-negative bacteria. Primers located within core genes *prfA* (CGSHiII_02915) and a putative transporting ATPase (CGSHiII_00679) were designed for the PCR amplification of SlrV Locus 1 based on the PittII genome (Bioproject #PRJNA16404). Sanger sequencing on an ABI 3730xl DNA analyzer was performed to determine gene sequences in PittII.

#### Bacterial strains and culture conditions

The bacterial strains used in this study are listed in Table 4. The NTHi strain PittII was recovered from a spontaneous pediatric otorrhea case [20]. The PittII mutant strains include the *msfA1-4* deletion mutant and an *msfA1* complement of the deletion mutant. The NTHi strain 86-028NP, recovered from the nasopharynx of a child with acute OM was also used [115]. We constructed an *msfA1-4* deletion mutant on the 86-028NP background as well. Further, we used the laboratory strain RdKW20 and a mutant that was engineered to produce *msfA1*_PittII_. All strains were cultivated in brain heart infusion (BHI) medium (Difco) supplemented with 10 μg mL-1 of hemin (ICN biochemicals) and 2 μg mL-1 NAD (Sigma); we refer to this medium as supplemented BHI (sBHI). For the inside-out staining in Fig 6B, we used PittII GFP, where GFP is transcribed from the prsm2211 plasmid which was obtained as a gift from Drs. Robert Munson and Lauren Bakaletz.

#### Strain Construction

DNA flanking the SlrV1 locus was amplified from PittII using primers 1/2 (flank1) and 3/4 (flank2) (S8 Table) and from 86-028NP using primers 5/6 (flank1) and 7/8 (flank2). Unique 5’ restriction sites were designed in primers to facilitate directional ligation. A kanamycin resistance cassette (km^R^) was amplified from the plasmid pHP1 using primers 9/10. Purified PCR products were digested using *NotI* and *SalI* restriction enzymes to generate non-complementary overhangs. Equimolar amounts of purified flank1, flank2 and km^R^ digests were ligated in a single reaction using T4 DNA ligase (New England Biolabs) with incubation overnight at 16°C. The ligation reactions were run on 0.6% TAE agarose gels and bands of the expected size were excised and purified. This purified gel cut ligation was used as template DNA in a PCR reaction using nested flanking primers, which were then purified and quantified using a Nanodrop 1000 Spectrophotometer and used as the transforming DNA. The *H*. *influenzae* complementation vector pASK5 described in Saeed-Kothe *et al* [116] was used to insert PittII *msfA1* into the *ompP1* locus of the SLR-KO strain as well as the Rd KW20 WT strain. This plasmid contains a multiple cloning site and a chloramphenicol resistance cassette (cm^R^) flanked by 5’ and 3’ regions of the nonessential *ompP1* gene. Amplification from constructs using this plasmid generates transforming DNA that inserts a gene of interest at the *ompP1* locus driven by the strong *ompP1* promoter. *MsfA1* was PCR amplified from PittII chromosomal DNA using primers 11/12 (S8 Table), purified, and digested with *BamHI* and *SalI* restriction enzymes. The *msfA1* fragment was ligated into *BamHI*/*SalI* digested pASK5 vector using T4 ligase. The empty vector was used to generate the PittII OMPP1-KO strain. Plasmid constructs were linearized and transformed into the PittII, PittII Msf-KO strain and Rd KW20 strain and transformants were selected by plating on sBHI plates containing chloramphenicol as described below.

#### Transformation Procedure

*H*. *influenzae* were grown at 37°C, shaking at 200 rpm in 5 mL of sBHI to log phase (OD_A600_ 0.4). 500 μL was transferred to a separate tube containing 1 μg of transforming DNA and mixed gently. The tube was incubated at 37°C for 10 minutes without shaking. 1 mL of pre-warmed sBHI was then added to each tube and incubated at 37°C for an additional 1.5 hours with shaking. 100 μL was then spread on multiple sBHI antibiotic plates. Km^r^ strains were selected by including kanamycin at 40 μg mL-1 and Cm^r^ strains were selected by including chloramphenicol at 2 μg mL-1. Plates were incubated at 37°C with 5% CO_2_ for 24 hours. Isolated colonies were picked into 5 mL sBHI with the appropriate antibiotic and incubated overnight at 37°C with shaking at 200 rpm. PCR reactions were performed using different combinations of primers listed in S8 Table from transformant and WT cultures to confirm the correct mutation had occurred. Positive cultures were frozen in 25% glycerol at -80°C.

#### Bacterial growth Assays

Starter cultures were grown to mid-log phase and used to inoculate 1 mL sBHI cultures in 24-well BD Falcon tissue-culture plates at an initial OD_A600_ of 0.02. Three wells in each plate containing media without bacterial inoculation were used to calculate background absorbance readings which were subtracted from each experimental well. Measurements were made on a Tecan Infinite M200 Pro plate reader set at 37°C with shaking at ~200 rpm. A script was programmed to take absorbance readings at 600 nm every 15 minutes. Data are representative of 3 biological replicates with n = 3 for each strain.

#### RNA isolation

Bacterial pellets stored in RNAProtect were resuspended in 100 μL of 1X Tris-EDTA (TE) + 1 mg mL-1 proteinase K (Qiagen). Tissues stored in RNALater were homogenized in RLT+ buffer. RNA was then extracted using a Qiagen RNeasy Mini Plus kit with the standard protocol including steps with genomic DNA (gDNA) eliminator columns. The eluted RNA (~85 μL) was DNased by adding 10 μL 10X TurboDNase buffer and 5 μL TurboDNase (2 units μL-1) (Ambion) and incubating at 37°C for 1.5 hours. 2 μL more TurboDNase was added and incubation continued for an additional 1.5 hours. The DNased RNA samples were cleaned by passing them through the RNeasy protocol a second time (including the gDNA eliminator column steps). Samples were eluted in nuclease free water, quantitated on a Nanodrop 1000 spectrophotometer and stored at -80°C. Each RNA sample was also run on an Agilent 2100 Bioanalyzer using RNA Nano6000 chips to check for RNA degradation.

#### Reverse transcription for gDNA check, microarrays and qRT-PCR

We performed paired reverse transcription reactions on every RNA sample where one reaction received reverse transcriptase (+RT, Promega) and the other did not (-RT). Both reactions were PCR amplified using primers directed against a housekeeping gene (*gapA*) and observation of amplification in the +RT reaction as well as lack of amplification in the -RT reaction verified removal of gDNA from each RNA sample. RNA for microarray analysis was reverse transcribed using a SuperScript One-Cycle cDNA Kit (Invitrogen) as outlined in the NimbleGen Microarray Experienced User’s Guide including RNaseA and cDNA precipitation steps. RNA for qRT-PCR was reverse transcribed using a Roche Transcriptor First Strand cDNA Synthesis kit with random hexamers.

#### HI Supragenome Hybridization (SGH) Array

A complete description of the design and methods associated with the HI SGH array for assessment of genic content are described by Eutsey *et al* [19]. Methods for performing microarray analysis with the HI SGH array are described in full by Janto *et al* [79]. Briefly, 1 μg of genomic DNA or cDNA was Cy3-labeled using a NimbleGen One-Color DNA Labeling Kit. NimbleGen Hybridization Kits and Sample Tracking Control Kits were used to hybridize the labeled cDNA to the custom-designed 4x72 *H*. *influenzae* SGH arrays as well as for array washing. Images were acquired on an Axon Instruments GenePix 4200AL array scanner. Images were processed and data was normalized within chips using a Robust Multichip Average (RMA) algorithm and quantile normalization via the NimbleScan software v2.5 [117, 118]. Raw data was converted into gene possession or absence by applying a combination of an expression threshold (1.5X the median background value in log_2_ scale) and a measure of probe variance [19]. Subclusters producing a signal above this value were set to a value of 1 (present) and subclusters with values below this value were set to a value of 0 (absent).

For microarray analysis raw expression data was merged with a reference list of genes/probes that had been determined to be present in the PittII genome (from SGH data) in order to remove non-relevant gene/probe data. Parsed data was then normalized within and across chips as described above. For comparison of PittII and Msf-KO expression data the web-based tool CyberT was used to obtain Bayesian corrected p-values, Bonferroni corrected p-values and Benjamini-Hochberg values [119]. Significance Analysis of Microarrays (SAM) in the ‘R’ environment was used to obtain lists of genes with associated permutation-based false discovery rates (FDR) [120]. These data were combined and filtered using the following cutoffs: Bayesian p-values < .05, Benjamini-Hochberg FDR < 10%, SAM local FDR < 0.1, SAM q-value < 0.1, Bonferroni corrected p-value < .05, average raw expression values in at least one of the two conditions being compared > 256. Only genes that passed all of these filters are presented in Table 5 and S6 Table. Data is representative of two biological replicates and two technical replicates. Raw and processed transcriptional data for this experiment has been deposited in NCBI's Gene Expression Omnibus (GEO) [80] and are accessible through GEO Series accession number GSE70172 (http://www.ncbi.nlm.nih.gov/geo/query/acc.cgi?acc=GSE70172).

#### Quantitative real-time polymerase chain reaction (qRT-PCR)

Gene specific primers were designed using Roche Probe Finder online software to generate ~75 bp amplicons (S7 Table). Amplification and quantitation was performed with a Roche Light Cycler 480 and SYBR green master mix. 20 μl reaction volumes were used containing 2 μl cDNA (1:5 dilution) and primers at 0.5 μM each. Relative expression levels of the tested genes were obtained by normalizing to the *gapA* reference gene as an internal standard. Each of two biological replicate RNA samples were assayed in duplicate. Data analysis was carried out using the Roche Light Cycler software. Data is representative of two biological replicates and three technical replicates.

#### Chinchilla Model of Otitis Media and Invasive Disease (OMID)

The comparisons of virulence between the WT and PittII Msf-KO strains were assessed as previously described [20]. All experiments were conducted with the approval of the Allegheny Singer Research Institute's Institutional Animal Care and Use Committee (IACUC). Young adult chinchillas (*Chinchilla laniger*, 400–600 gm; McClenahan Chinchilla Ranch, New Wilmington, PA) were obtained free of middle-ear disease as culls from the fur industry. After at least a 7-day acclimation period, the animals were anesthetized on experimental day 0 by intramuscular injection of 0.1 mL of a cocktail of ketamine hydrochloride 100 mg mL-1, xylazine hydrochloride 30 mg mL-1 and acepromazine 5 mg mL-1. After anesthesia was confirmed (abolishment of eye-blink reflex) bacteria were injected bilaterally through the tympanic bullae using a 0.5 inch, 27-gauge needle on a 1 mL tuberculin syringe into the middle ear space of chinchillas as described [20]. Three experiments were performed with differing numbers of animals, inoculum amounts and/or inoculum preparations. 1) To determine whether m*sfA* was transcribed *in vivo*, one animal was inoculated bilaterally (into each ear) with 10^8^ CFU of PittII WT and was euthanized 3 hours later. One animal was inoculated bilaterally with 10^6^ CFU of PittII WT and euthanized after 24 hours. The middle-ear mucosa with the adherent bacterial biofilm as well as lavages were harvested into RNA-later. RNA was extracted as described above. 2) For the *in vivo* competition experiment 10^3^ CFU of a mixed culture (WT:Msf-KO or WT:Msf-COMP) was injected bilaterally into the middle ears of six chinchillas. Animals were euthanized on day three for tissue collection. The right and left middle-ear mucosa with the adherent bacterial biofilm (Bullar membrane), lavages from both ears (Bullar effusion), brains and lungs were collected, homogenized and plated for bacterial counts. 3) For the *in vivo* virulence experiment comparing separate PittII and Msf-KO infections, 10^3^ CFU of PittII were injected bilaterally into the middle ears of 10 chinchillas and 10^3^ CFU of Msf-KO were injected into the middle ears of 9 chinchillas (1 animal was lost prior to the beginning of the experiment). The animals were monitored daily for twelve days for signs and severity of local (otologic) and systemic disease using the criteria in S5 Table. Any animal that was determined to have symptoms corresponding to a systemic score of 4 was euthanized immediately. Animals that did not succumb to infection were euthanized at day 12 for tissue collection. All evaluations were performed by an observer who was blinded with regard to the inoculating strains. Local disease was evaluated by a single validated otoscopist (a practicing board-certified, fellowship-trained, otolaryngological surgeon) to ensure uniformity. Hence for each animal three scores were recorded daily: otoscopic score for right ear, otoscopic score for left ear and systemic score. From the collected data we also evaluated measures relating to the severity of local disease: maximum otologic score, days to first significant otologic score, and days to maximum otologic score. We also determined measures relating to systemic evaluations including rapidity of onset, maximum severity of disease, and mortality. As soon as possible after death, animals were dissected. The right and left middle-ear mucosa as well as lavages or each middle-ear, lungs and brains were harvested and homogenized. The homogenates were serially diluted and plated to determine the presence of infecting strains.

#### *In vitro* competition experiments

Mixed-culture experiments were performed (**a**) in planktonic culture (**b**) in *in vitro* biofilms. Broth starter cultures were grown to mid-log phase and cultures were adjusted such that equal numbers of bacteria were mixed together to a final concentration of 10^3^ cfu mL-1. For planktonic competition experiments (n = 3) mixed cultures (n = 3) were grown in 15 mL culture tubes at 37°C, shaking at 200 rpm. At time 0 and 24 hours, cultures were serially diluted and plated on two sets of plates: sBHI (for total bacterial count) and sBHI+km 40 μg mL-1 (for PittII Msf-KO count). A competitive index was calculated with the formula: (KO_t = 24_ / WT_t = 24_) / (KO_t = 0_ / WT_t = 0_). For *in vitro* static biofilm competition experiments (n = 3) mixed cultures were seeded into 6 well culture plates and initially incubated at 37°C with 5% CO_2_ (v/v) without shaking. After 2 hours the plates were set to rotate slowly at 50 rpm. At each time point (0, 24, 48, and 72 hours) three replicate wells were harvested by first collecting the supernatant (media in the wells plus two PBS washes). Following washing, biofilms were mechanically disrupted in PBS using a cell scraper, collected and washed/disrupted a second time. Both collection samples (supernatant and biofilm) were vortexed vigorously and were serially diluted and plated on two sets of plates as described above.

#### Macrophage survival assays

The human monocyte cell line THP-1 (ATCC TIB-202) was maintained in RPMI media (ATCC) supplemented with 10% (v/v) fetal bovine serum (FBS) (ATCC) and 0.05 mM 2-mercaptoethanol (Sigma). The cells were maintained as monocyte-like, non-adherent cells at 37°C with 5% (v/v) CO_2_. For macrophage infection, cells were seeded at 5 X 10^5^ cells per well in 24 well tissue culture plates and were differentiated by addition of phorbol 12-myristate 13-acetate (PMA) (1 μg mL-1) for 24 hours. After 24 hours fresh media containing PMA was added. After another 24 hours the medium was then removed and the macrophages were infected with stationary phase cultures of bacterial strains that had been diluted in RPMI + 10% FBS to achieve a multiplicity of infection (MOI) of 100 bacteria per macrophage. Plates were centrifuged for 15 minutes at 200 x g at room temperature and then incubated at 37°C with 5% (v/v) CO_2_ for 1 hour. Next, the macrophages were washed twice and fresh media with 10 μg mL-1 of polymyxin B was added. At 2, 24, 48 and 72 hours post inoculation, wells of infected macrophages were washed twice and then lysed with 1% saponin (MP Biomedicals, LLC) in PBS. Serial dilutions of the resulting macrophage lysates were plated onto sBHI plates for CFU counts. Data is representative of 3 experiments with n = 3 for each strain. In the inhibition experiments, prior to the addition of PMA, cells were pretreated with Cytochalasin D (1 μM) for 1 hour (Sigma Chemical Co. St. Louis, MO, USA).

#### Inside Out Staining for Confocal Imaging

THP-1 monocytes were seeded into plates, differentiated, and then infected with PittII GFP as described above. After one hour, polymyxin B was added to each well to kill extracellular bacteria (see survival protocol). At designated time points (2, 24, 48 and 72 hours) the macrophage monolayers were washed twice with PBS and then fixed with 4% paraformaldehyde (PFA) for 30 minutes. PFA was removed and the cells were again washed with PBS twice. For storage the fixed cells were kept in 50% ethanol/50% PBS at 4°C. For staining, samples were blocked with 10% FBS for 1 hour at room temperature. Any remaining extracellular bacteria were stained using a rabbit anti-NTHi antiserum (obtained as a gift from Dr. Ed Swords, Wake Forest University) and Alexa Fluor 594-conjugated anti-rabbit antibodies (Biotium). Confocal images were obtained and analyzed on a Leica TCS SP2 AOBS filter free spectral confocal microscopy system. Bacteria outside macrophages appeared either red (dead) or yellow (alive and expressing GFP mixed with signal from the antibody stains) whereas bacteria inside macrophages appeared green (alive and expressing GFP with no antibody stain).

#### Statistical Testing

All statistical tests were carried out in the R statistical environment (version 3.1.1).

For *in vitro* competition data (Fig 7A and 7B) the CI values were log transformed and evaluated by one-sample two-tailed *t*-tests using μ_0_ = 0 and α = 0.05.

For *in vitro* macrophage survival data the percentage of surviving bacteria was logit transformed for subsequent statistical testing. The PittII experiment (Fig 6A) with four strains (WT, Msf-KO, Msf-COMP and OMPP1-KO) was evaluated by a weighted One-Way analysis of variance (ANOVA) test for independent samples using α = 0.05 at each time-point. Tests which rejected the null-hypothesis (p<0.05) were further tested with the Tukey HSD post-hoc test using α = 0.05. For the 86-028NP and Rd KW20 experiments (Fig 6C and 6D), WT and mutant pairs were evaluated with unpaired two-tailed *t*-tests using α = 0.05 at each time-point.

For *in vivo* competition data (Fig 7D) raw CFU values were log transformed and evaluated by unpaired two-tailed *t*-tests using α = 0.05 for each strain pair from each tissue. WT:Msf-KO and WT:Msf-COMP data sets from each tissue site were further evaluated by the Mann-Whitney U test using α = 0.05.

For evaluation of data involving presence/absence of bacteria including the *in vivo* competition infection experiment (Fig 7E) and the *in vivo* single strain infection experiments (Fig 8B), two-tailed Fisher-Exact tests using α = 0.05 were performed.

## Supporting Information

S1 Fig *SlrV* motifs found in *H*. *influenzae*. Sequence logos based on MEME/MAST analysis that represent the 9 *slrV* genes. Motifs were generated using the R Bioconductor package motifStack. Amino acid colors are a modification of the WebLogo default, with Tyr and Cys having unique colors (Y = orange and C = turquoise). The number of sequences each logo is based on is indicated above each panel as n = #. Arrow indicates the location of the 100% conserved tyrosine residue.
(TIFF)

S2 Fig Broth growth curves of PittII WT, Msf-KO and Msf-COMP. Planktonic growth in BHI broth of 1 mL cultures in 24-well plates.
(TIFF)

S3 Fig Phylogenetic inference from SLR protein domains (rectangular). 36 amino acid SLR motifs extracted from the 79 genes containing them (n=256). Motifs found within the same protein do not cluster together. Node labels indicate the strain, strain-specific SGH cluster ID number, and location of the motif within the CDS (amino acid position). This tree corresponds to Fig 3D.
(TIFF)

S1 Table List of SLR-containing *H*. *influenzae* ORFs identified from MEME/MAST analysis. (XLSX)

S2 Table Consensus motifs found in *H*. *influenzae* SLR-containing protein subfamilies. Each consensus motif was defined by the most prevalent amino acid found at each position in the motif. n = number of sequences used to generate each motif.
(XLSX)

S3 Table Distribution of SLR-containing genes for 210 H. influenzae strains. Columns B-K are gene possession data from the supragenome hybridization array; "1" presence and "0" absence of each listed gene. Columns L-M are data from PCR analysis of each strain. # based on PCR using primers to genes upstream and downstream of *slrC*. * based on PCR with primers to the putative transporting ATPase and *prfA*; size in the absence of *slrV* genes is ~1300 bp.
(XLSX)

S4 Table Predicted amino acid sequences of PittII SlrVA (Msf) proteins. (XLSX)

S5 Table *Chinchilla laniger* model of otitis media and invasive disease (OMID) virulence scoring system. (XLSX)

S6 Table Genes differentially regulated in the PittII Msf-KO strain compared to the WT strain. Genes that passed all filters based on SAM q-values, SAM and Benjamini-Hochberg (BH) false discovery rates (FDR), p-values (Bon: Bonferroni-corrected) and raw expression cutoffs. WT-avg: Average expression value for PittII. KO-avg: Average expression value for PittII Msf-KO. Genes are sorted by the magnitude of fold changes.
(XLSX)

S7 Table Primers used for qRT-PCR. (XLSX)

S8 Table Primers used in strain construction. (XLSX)

## References

[1] MacNeil JR, Cohn AC, Farley M, Mair R, Baumbach J, Bennett N, et al Current epidemiology and trends in invasive Haemophilus influenzae disease—United States, 1989–2008. Clin Infect Dis. 2011;53(12):1230–6. 10.1093/cid/cir735 22080119

[2] Post JC, Preston RA, Aul JJ, Larkins-Pettigrew M, Rydquist-White J, Anderson KW, et al Molecular analysis of bacterial pathogens in otitis media with effusion. JAMA. 1995;273(20):1598–604. 7745773

[3] Ehrlich GD, Veeh R, Wang X, Costerton JW, Hayes JD, Hu FZ, et al Mucosal biofilm formation on middle-ear mucosa in the chinchilla model of otitis media. JAMA. 2002;287(13):1710–5. 11926896 10.1001/jama.287.13.1710

[4] Hall-Stoodley L, Hu FZ, Gieseke A, Nistico L, Nguyen D, Hayes J, et al Direct detection of bacterial biofilms on the middle-ear mucosa of children with chronic otitis media. JAMA. 2006;296(2):202–11. 16835426 10.1001/jama.296.2.202 PMC1885379

[5] Brook I, Yocum P, Shah K. Aerobic and anaerobic bacteriology of otorrhea associated with tympanostomy tubes in children. Acta Otolaryngol. 1998;118(2):206–10. 9583788 10.1080/00016489850154919

[6] Dohar JE, Hebda PA, Veeh R, Awad M, Costerton JW, Hayes J, et al Mucosal biofilm formation on middle-ear mucosa in a nonhuman primate model of chronic suppurative otitis media. Laryngoscope. 2005;115(8):1469–72. 16094127 10.1097/01.mlg.0000172036.82897.d4

[7] Sanderson AR, Leid JG, Hunsaker D. Bacterial biofilms on the sinus mucosa of human subjects with chronic rhinosinusitis. Laryngoscope. 2006;116(7):1121–6. 16826045 10.1097/01.mlg.0000221954.05467.54

[8] Murphy TF. Respiratory infections caused by non-typeable Haemophilus influenzae. Curr Opin Infect Dis. 2003;16(2):129–34. 12734445 10.1097/00001432-200304000-00009

[9] Livorsi DJ, Macneil JR, Cohn AC, Bareta J, Zansky S, Petit S, et al Invasive Haemophilus influenzae in the United States, 1999–2008: epidemiology and outcomes. J Infect. 2012;65(6):496–504. 10.1016/j.jinf.2012.08.005 22902945 PMC4329643

[10] Strouts FR, Power P, Croucher NJ, Corton N, van Tonder A, Quail MA, et al Lineage-specific virulence determinants of Haemophilus influenzae biogroup aegyptius. Emerg Infect Dis. 2012;18(3):449–57. 10.3201/eid1803.110728 22377449 PMC3309571

[11] Tsang RS, Sill ML, Skinner SJ, Law DK, Zhou J, Wylie J. Characterization of invasive Haemophilus influenzae disease in Manitoba, Canada, 2000–2006: invasive disease due to non-type b strains. Clin Infect Dis. 2007;44(12):1611–4. 17516405 10.1086/518283

[12] Shuel M, Law D, Skinner S, Wylie J, Karlowsky J, Tsang RS. Characterization of nontypeable Haemophilus influenzae collected from respiratory infections and invasive disease cases in Manitoba, Canada. FEMS Immunol Med Microbiol. 2010;58(2):277–84. 10.1111/j.1574-695X.2009.00634.x 20041949

[13] Kelly L, Tsang RS, Morgan A, Jamieson FB, Ulanova M. Invasive disease caused by Haemophilus influenzae type a in Northern Ontario First Nations communities. J Med Microbiol. 2011;60(Pt 3):384–90. 10.1099/jmm.0.026914-0 21148281

[14] Dworkin MS, Park L, Borchardt SM. The changing epidemiology of invasive Haemophilus influenzae disease, especially in persons > or = 65 years old. Clin Infect Dis. 2007;44(6):810–6. 17304452 10.1086/511861

[15] Resman F, Ristovski M, Ahl J, Forsgren A, Gilsdorf JR, Jasir A, et al Invasive disease caused by Haemophilus influenzae in Sweden 1997–2009; evidence of increasing incidence and clinical burden of non-type b strains. Clin Microbiol Infect. 2011;17(11):1638–45. 10.1111/j.1469-0691.2010.03417.x 21054663

[16] Wan Sai Cheong J, Smith H, Heney C, Robson J, Schlebusch S, Fu J, et al Trends in the epidemiology of invasive Haemophilus influenzae disease in Queensland, Australia from 2000 to 2013: what is the impact of an increase in invasive non-typable H. influenzae (NTHi)? Epidemiol Infect. 2015;143(14):2993–3000. 10.1017/S0950268815000345 25762194 PMC9151033

[17] Hogg J, Hu F, Janto B, Boissy R, Hayes J, Keefe R, et al Characterization and modeling of the Haemophilus influenzae core and supragenomes based on the complete genomic sequences of Rd and 12 clinical nontypeable strains. Genome Biol. 2007;8(6):R103 17550610 10.1186/gb-2007-8-6-r103 PMC2394751

[18] Boissy R, Ahmed A, Janto B, Earl J, Hall BG, Hogg JS, et al Comparative supragenomic analyses among the pathogens Staphylococcus aureus, Streptococcus pneumoniae, and Haemophilus influenzae using a modification of the finite supragenome model. BMC Genomics. 2011;12:187 10.1186/1471-2164-12-187 21489287 PMC3094309

[19] Eutsey RA, Hiller NL, Earl JP, Janto BA, Dahlgren ME, Ahmed A, et al Design and validation of a supragenome array for determination of the genomic content of Haemophilus influenzae isolates. BMC Genomics. 2013;14:484 10.1186/1471-2164-14-484 23865594 PMC3723446

[20] Buchinsky FJ, Forbes ML, Hayes JD, Shen K, Ezzo S, Compliment J, et al Virulence phenotypes of low-passage clinical isolates of nontypeable Haemophilus influenzae assessed using the chinchilla laniger model of otitis media. BMC Microbiol. 2007;7:56 17570853 10.1186/1471-2180-7-56 PMC1914350

[21] Ehrlich GD, Ahmed A, Earl J, Hiller NL, Costerton JW, Stoodley P, et al The distributed genome hypothesis as a rubric for understanding evolution in situ during chronic bacterial biofilm infectious processes. FEMS Immunol Med Microbiol. 2010;59(3):269–79. 10.1111/j.1574-695X.2010.00704.x 20618850 PMC2910629

[22] Gilsdorf JR, Marrs CF, Foxman B. Haemophilus influenzae: genetic variability and natural selection to identify virulence factors. Infect Immun. 2004;72(5):2457–61. 15102751 10.1128/IAI.72.5.2457-2461.2004 PMC387884

[23] Murphy TF. Vaccines for Nontypeable Haemophilus influenzae. The Future is Now. Clin Vaccine Immunol. 2015. 10.1128/CVI.00089-15 PMC4412935 25787137

[24] Dagan R, Leibovitz E, Cheletz G, Leiberman A, Porat N. Antibiotic treatment in acute Otitis Media promotes superinfection with resistant Streptococcus pneumoniae carried before initiation of treatment. J Infect Dis. 2001;183(6):880–6. 11237804 10.1086/319250

[25] Klein JO. Clinical implications of antibiotic resistance for management of acute otitis media. The Journal of laboratory and clinical medicine. 2000;135(3):220–4. 10711859 10.1067/mlc.2000.105614

[26] Bondy J, Berman S, Glazner J, Lezotte D. Direct expenditures related to otitis media diagnoses: extrapolations from a pediatric medicaid cohort. Pediatrics. 2000;105(6):E72 10835085 10.1542/peds.105.6.e72

[27] Block SL, Harrison CJ, Hedrick J, Tyler R, Smith A, Hedrick R. Restricted use of antibiotic prophylaxis for recurrent acute otitis media in the era of penicillin non-susceptible Streptococcus pneumoniae. International journal of pediatric otorhinolaryngology. 2001;61(1):47–60. 11576631 10.1016/s0165-5876(01)00550-x

[28] Geme JW St 3rd. Molecular and cellular determinants of non-typeable Haemophilus influenzae adherence and invasion. Cell Microbiol. 2002;4(4):191–200. 11952636 10.1046/j.1462-5822.2002.00180.x

[29] Mason KM, Munson RS Jr., Bakaletz LO. A mutation in the sap operon attenuates survival of nontypeable Haemophilus influenzae in a chinchilla model of otitis media. Infect Immun. 2005;73(1):599–608. 15618200 10.1128/IAI.73.1.599-608.2005 PMC538956

[30] Sikkema DJ, Murphy TF. Molecular analysis of the P2 porin protein of nontypeable Haemophilus influenzae. Infect Immun. 1992;60(12):5204–11. 1280627 10.1128/iai.60.12.5204-5211.1992 PMC258298

[31] Duim B, van Alphen L, Eijk P, Jansen HM, Dankert J. Antigenic drift of non-encapsulated Haemophilus influenzae major outer membrane protein P2 in patients with chronic bronchitis is caused by point mutations. Mol Microbiol. 1994;11(6):1181–9. 8022287 10.1111/j.1365-2958.1994.tb00394.x

[32] Brunham RC, Plummer FA, Stephens RS. Bacterial antigenic variation, host immune response, and pathogen-host coevolution. Infect Immun. 1993;61(6):2273–6. 8500868 10.1128/iai.61.6.2273-2276.1993 PMC280844

[33] Gilsdorf JR. Antigenic Diversity and Gene Polymorphisms in Haemophilus influenzae. Infect Immun. 1998;66(11):5053–9. 9784503 10.1128/iai.66.11.5053-5059.1998 PMC108629

[34] Swords WE, Jones PA, Apicella MA. The lipo-oligosaccharides of Haemophilus influenzae: an interesting array of characters. J Endotoxin Res. 2003;9(3):131–44. 12831454 10.1179/096805103125001531

[35] Hood DW, Deadman ME, Jennings MP, Bisercic M, Fleischmann RD, Venter JC, et al DNA repeats identify novel virulence genes in Haemophilus influenzae. Proc Natl Acad Sci U S A. 1996;93(20):11121–5. 8855319 10.1073/pnas.93.20.11121 PMC38294

[36] Forsgren J, Samuelson A, Ahlin A, Jonasson J, Rynnel-Dagoo B, Lindberg A. Haemophilus influenzae resides and multiplies intracellularly in human adenoid tissue as demonstrated by in situ hybridization and bacterial viability assay. Infect Immun. 1994;62(2):673–9. 7507900 10.1128/iai.62.2.673-679.1994 PMC186156

[37] Forsgren J, Samuelson A, Borrelli S, Christensson B, Jonasson J, Lindberg AA. Persistence of nontypeable Haemophilus influenzae in adenoid macrophages: a putative colonization mechanism. Acta Otolaryngol. 1996;116(5):766–73. 8908258 10.3109/00016489609137922

[38] Swords WE, Buscher BA, Ver Steeg Ii K, Preston A, Nichols WA, Weiser JN, et al Non-typeable Haemophilus influenzae adhere to and invade human bronchial epithelial cells via an interaction of lipooligosaccharide with the PAF receptor. Mol Microbiol. 2000;37(1):13–27. 10931302 10.1046/j.1365-2958.2000.01952.x

[39] Swords WE, Ketterer MR, Shao J, Campbell CA, Weiser JN, Apicella MA. Binding of the non-typeable Haemophilus influenzae lipooligosaccharide to the PAF receptor initiates host cell signalling. Cell Microbiol. 2001;3(8):525–36. 11488814 10.1046/j.1462-5822.2001.00132.x

[40] Morey P, Cano V, Marti-Lliteras P, Lopez-Gomez A, Regueiro V, Saus C, et al Evidence for a non-replicative intracellular stage of nontypable Haemophilus influenzae in epithelial cells. Microbiology. 2011;157(Pt 1):234–50. 10.1099/mic.0.040451-0 20929955

[41] Craig JE, Cliffe A, Garnett K, High NJ. Survival of nontypeable Haemophilus influenzae in macrophages. FEMS Microbiol Lett. 2001;203(1):55–61. 11557140 10.1111/j.1574-6968.2001.tb10820.x

[42] Ahren IL, Janson H, Forsgren A, Riesbeck K. Protein D expression promotes the adherence and internalization of non-typeable Haemophilus influenzae into human monocytic cells. Microb Pathog. 2001;31(3):151–8. 11500100 10.1006/mpat.2001.0456

[43] Ahren IL, Williams DL, Rice PJ, Forsgren A, Riesbeck K. The importance of a beta-glucan receptor in the nonopsonic entry of nontypeable Haemophilus influenzae into human monocytic and epithelial cells. J Infect Dis. 2001;184(2):150–8. 11424011 10.1086/322016

[44] Farley MM, Stephens DS, Mulks MH, Cooper MD, Bricker JV, Mirra SS, et al Pathogenesis of IgA1 protease-producing and -nonproducing Haemophilus influenzae in human nasopharyngeal organ cultures. J Infect Dis. 1986;154(5):752–9. 3534106 10.1093/infdis/154.5.752

[45] Bandi V, Apicella MA, Mason E, Murphy TF, Siddiqi A, Atmar RL, et al Nontypeable Haemophilus influenzae in the lower respiratory tract of patients with chronic bronchitis. Am J Respir Crit Care Med. 2001;164(11):2114–9. 11739144 10.1164/ajrccm.164.11.2104093

[46] Ketterer MR, Shao JQ, Hornick DB, Buscher B, Bandi VK, Apicella MA. Infection of primary human bronchial epithelial cells by Haemophilus influenzae: macropinocytosis as a mechanism of airway epithelial cell entry. Infect Immun. 1999;67(8):4161–70. 10417188 10.1128/iai.67.8.4161-4170.1999 PMC96721

[47] Geme JW St, Falkow S. Haemophilus influenzae adheres to and enters cultured human epithelial cells. Infect Immun. 1990;58(12):4036–44. 2254028 10.1128/iai.58.12.4036-4044.1990 PMC313773

[48] Hotomi M, Arai J, Billal DS, Takei S, Ikeda Y, Ogami M, et al Nontypeable Haemophilus influenzae isolated from intractable acute otitis media internalized into cultured human epithelial cells. Auris Nasus Larynx. 2010;37(2):137–44. 10.1016/j.anl.2009.03.012 19505782

[49] Clementi CF, Murphy TF. Non-Typeable Haemophilus influenzae Invasion and Persistence in the Human Respiratory Tract. Front Cell Infect Microbiol. 2011;1. 10.3389/fcimb.2011.00001 PMC3417339 22919570

[50] Williams AE, Maskell DJ, Moxon ER. Relationship between intracellular survival in macrophages and virulence of Haemophilus influenzae type b. J Infect Dis. 1991;163(6):1366–9. 2037802 10.1093/infdis/163.6.1366

[51] Cieri MV, Mayer-Hamblett N, Griffith A, Burns JL. Correlation between an In Vitro Invasion Assay and a Murine Model of Burkholderia cepacia Lung Infection. Infect Immun. 2002;70(3):1081–6. 11854186 10.1128/IAI.70.3.1081-1086.2002 PMC127769

[52] Martin DW, Mohr CD. Invasion and intracellular survival of Burkholderia cepacia. Infect Immun. 2000;68(1):24–9. 10603364 10.1128/iai.68.1.24-29.2000 PMC97097

[53] Uhlich GA, Keen JE, Elder RO. Variations in the csgD promoter of Escherichia coli O157:H7 associated with increased virulence in mice and increased invasion of HEp-2 cells. Infect Immun. 2002;70(1):395–9. 11748206 10.1128/IAI.70.1.395-399.2002 PMC127602

[54] Norton PM, Rolph C, Ward PN, Bentley RW, Leigh JA. Epithelial invasion and cell lysis by virulent strains of Streptococcus suis is enhanced by the presence of suilysin. FEMS Immunol Med Microbiol. 1999;26(1):25–35. 10518040 10.1111/j.1574-695X.1999.tb01369.x

[55] Read TD, Satola SW, Opdyke JA, Farley MM. Copy number of pilus gene clusters in Haemophilus influenzae and variation in the hifE pilin gene. Infect Immun. 1998;66(4):1622–31. 9529090 10.1128/iai.66.4.1622-1631.1998 PMC108097

[56] Lindenthal C, Elsinghorst EA. Identification of a Glycoprotein Produced by Enterotoxigenic Escherichia coli. Infect Immun. 1999;67(8):4084–91. 10417177 10.1128/iai.67.8.4084-4091.1999 PMC96707

[57] Henderson IR, Nataro JP. Virulence Functions of Autotransporter Proteins. Infect Immun. 2001;69(3):1231–43. 11179284 10.1128/IAI.69.3.1231-1243.2001 PMC98013

[58] Bailey TL, Boden M, Buske FA, Frith M, Grant CE, Clementi L, et al MEME SUITE: tools for motif discovery and searching. Nucleic Acids Res. 2009;37(Web Server issue):W202–8. 10.1093/nar/gkp335 19458158 PMC2703892

[59] Bailey TL, Elkan C. Fitting a mixture model by expectation maximization to discover motifs in biopolymers. Proc Int Conf Intell Syst Mol Biol. 1994;2:28–36. 7584402

[60] Bailey TL, Gribskov M. Combining evidence using p-values: application to sequence homology searches. Bioinformatics. 1998;14(1):48–54. 9520501 10.1093/bioinformatics/14.1.48

[61] Cirillo SL, Lum J, Cirillo JD. Identification of novel loci involved in entry by Legionella pneumophila. Microbiology. 2000;146 (Pt 6):1345–59. 10846213 10.1099/00221287-146-6-1345

[62] Newton HJ, Sansom FM, Bennett-Wood V, Hartland EL. Identification of Legionella pneumophila-specific genes by genomic subtractive hybridization with Legionella micdadei and identification of lpnE, a gene required for efficient host cell entry. Infect Immun. 2006;74(3):1683–91. 16495539 10.1128/IAI.74.3.1683-1691.2006 PMC1418643

[63] Newton HJ, Sansom FM, Dao J, McAlister AD, Sloan J, Cianciotto NP, et al Sel1 repeat protein LpnE is a Legionella pneumophila virulence determinant that influences vacuolar trafficking. Infect Immun. 2007;75(12):5575–85. 17893138 10.1128/IAI.00443-07 PMC2168337

[64] Liu M, Conover GM, Isberg RR. Legionella pneumophila EnhC is required for efficient replication in tumour necrosis factor alpha-stimulated macrophages. Cell Microbiol. 2008;10(9):1906–23. 10.1111/j.1462-5822.2008.01180.x 18549456 PMC2579332

[65] Weber SS, Ragaz C, Hilbi H. The inositol polyphosphate 5-phosphatase OCRL1 restricts intracellular growth of Legionella, localizes to the replicative vacuole and binds to the bacterial effector LpnE. Cell Microbiol. 2009;11(3):442–60. 10.1111/j.1462-5822.2008.01266.x 19021631

[66] Liu M, Haenssler E, Uehara T, Losick VP, Park JT, Isberg RR. The Legionella pneumophila EnhC protein interferes with immunostimulatory muramyl peptide production to evade innate immunity. Cell Host Microbe. 2012;12(2):166–76. 10.1016/j.chom.2012.06.004 22901537 PMC3678716

[67] Deml L, Aigner M, Decker J, Eckhardt A, Schutz C, Mittl PR, et al Characterization of the Helicobacter pylori cysteine-rich protein A as a T-helper cell type 1 polarizing agent. Infect Immun. 2005;73(8):4732–42. 16040986 10.1128/IAI.73.8.4732-4742.2005 PMC1201243

[68] Ogura M, Perez JC, Mittl PR, Lee HK, Dailide G, Tan S, et al Helicobacter pylori evolution: lineage- specific adaptations in homologs of eukaryotic Sel1-like genes. PLoS Comput Biol. 2007;3(8):e151 17696605 10.1371/journal.pcbi.0030151 PMC1941758

[69] Dumrese C, Slomianka L, Ziegler U, Choi SS, Kalia A, Fulurija A, et al The secreted Helicobacter cysteine-rich protein A causes adherence of human monocytes and differentiation into a macrophage-like phenotype. FEBS Lett. 2009;583(10):1637–43. 10.1016/j.febslet.2009.04.027 19393649 PMC2764743

[70] Roschitzki B, Schauer S, Mittl PR. Recognition of host proteins by Helicobacter cysteine-rich protein C. Curr Microbiol. 2011;63(3):239–49. 10.1007/s00284-011-9969-2 21735226

[71] Mittl PR, Luthy L, Hunziker P, Grutter MG. The cysteine-rich protein A from Helicobacter pylori is a beta-lactamase. J Biol Chem. 2000;275(23):17693–9. 10748053 10.1074/jbc.M001869200

[72] Luthy L, Grutter MG, Mittl PR. The crystal structure of Helicobacter pylori cysteine-rich protein B reveals a novel fold for a penicillin-binding protein. J Biol Chem. 2002;277(12):10187–93. 11777911 10.1074/jbc.M108993200

[73] Chong A, Child R, Wehrly TD, Rockx-Brouwer D, Qin A, Mann BJ, et al Structure-Function Analysis of DipA, a Virulence Factor Required for Intracellular Replication. PLoS One. 2013;8(6):e67965 23840797 10.1371/journal.pone.0067965 PMC3694160

[74] Wehrly TD, Chong A, Virtaneva K, Sturdevant DE, Child R, Edwards JA, et al Intracellular biology and virulence determinants of Francisella tularensis revealed by transcriptional profiling inside macrophages. Cell Microbiol. 2009;11(7):1128–50. 10.1111/j.1462-5822.2009.01316.x 19388904 PMC2746821

[75] Pastorello I, Rossi Paccani S, Rosini R, Mattera R, Ferrer Navarro M, Urosev D, et al EsiB, a novel pathogenic Escherichia coli secretory immunoglobulin A-binding protein impairing neutrophil activation. MBio. 2013;4(4). 10.1128/mBio.00206-13 PMC3735183 23882011

[76] Urosev D, Ferrer-Navarro M, Pastorello I, Cartocci E, Costenaro L, Zhulenkovs D, et al Crystal structure of c5321: a protective antigen present in uropathogenic Escherichia coli strains displaying an SLR fold. BMC Struct Biol. 2013;13:19 10.1186/1472-6807-13-19 24099525 PMC3851747

[77] Zhang L, Foxman B, Manning SD, Tallman P, Marrs CF. Molecular Epidemiologic Approaches to Urinary Tract Infection Gene Discovery in Uropathogenic Escherichia coli. Infect Immun. 2000;68(4):2009–15. 10722596 10.1128/iai.68.4.2009-2015.2000 PMC97380

[78] Pettigrew MM, Foxman B, Marrs CF, Gilsdorf JR. Identification of the Lipooligosaccharide Biosynthesis Gene lic2B as a Putative Virulence Factor in Strains of Nontypeable Haemophilus influenzae That Cause Otitis Media. Infect Immun. 2002;70(7):3551–6. 12065495 10.1128/IAI.70.7.3551-3556.2002 PMC128108

[79] Janto BA, Hiller NL, Eutsey RA, Dahlgren ME, Earl JP, Powell E, et al Development and validation of an Haemophilus influenzae supragenome hybridization (SGH) array for transcriptomic analyses. PLoS One. 2014;9(10):e105493 10.1371/journal.pone.0105493 25290153 PMC4188559

[80] Edgar R, Domrachev M, Lash AE. Gene Expression Omnibus: NCBI gene expression and hybridization array data repository. Nucleic acids research. 2002;30(1):207–10. 11752295 10.1093/nar/30.1.207 PMC99122

[81] Pittman MS, Kelly DJ. Electron transport through nitrate and nitrite reductases in Campylobacter jejuni. Biochem Soc Trans. 2005;33(Pt 1):190–2. 15667303 10.1042/BST0330190

[82] Potter L, Angove H, Richardson D, Cole J. Nitrate reduction in the periplasm of gram-negative bacteria. Adv Microb Physiol. 2001;45:51–112. 11450112 10.1016/s0065-2911(01)45002-8

[83] Poock SR, Leach ER, Moir JW, Cole JA, Richardson DJ. Respiratory detoxification of nitric oxide by the cytochrome c nitrite reductase of Escherichia coli. The Journal of biological chemistry. 2002;277(26):23664–9. 11960983 10.1074/jbc.M200731200

[84] Thony-Meyer L. Haem-polypeptide interactions during cytochrome c maturation. Biochimica et biophysica acta. 2000;1459(2–3):316–24. 11004446 10.1016/s0005-2728(00)00167-5

[85] Tanapongpipat S, Reid E, Cole JA, Crooke H. Transcriptional control and essential roles of the Escherichia coli ccm gene products in formate-dependent nitrite reduction and cytochrome c synthesis. The Biochemical journal. 1998;334 (Pt 2):355–65. 9716493 10.1042/bj3340355 PMC1219697

[86] Weiner JH, MacIsaac DP, Bishop RE, Bilous PT. Purification and properties of Escherichia coli dimethyl sulfoxide reductase, an iron-sulfur molybdoenzyme with broad substrate specificity. J Bacteriol. 1988;170(4):1505–10. 3280546 10.1128/jb.170.4.1505-1510.1988 PMC210994

[87] Cammack R, Weiner JH. Electron paramagnetic resonance spectroscopic characterization of dimethyl sulfoxide reductase of Escherichia coli. Biochemistry. 1990;29(36):8410–6. 2174699 10.1021/bi00488a030

[88] Cotter PA, Gunsalus RP. Oxygen, nitrate, and molybdenum regulation of dmsABC gene expression in Escherichia coli. J Bacteriol. 1989;171(7):3817–23. 2544558 10.1128/jb.171.7.3817-3823.1989 PMC210130

[89] Gon S, Patte JC, Mejean V, Iobbi-Nivol C. The torYZ (yecK bisZ) operon encodes a third respiratory trimethylamine N-oxide reductase in Escherichia coli. J Bacteriol. 2000;182(20):5779–86. 11004177 10.1128/jb.182.20.5779-5786.2000 PMC94700

[90] Berks BC, Ferguson SJ, Moir JW, Richardson DJ. Enzymes and associated electron transport systems that catalyse the respiratory reduction of nitrogen oxides and oxyanions. Biochim Biophys Acta. 1995;1232(3):97–173. 8534676 10.1016/0005-2728(95)00092-5

[91] Bayles KW. The bactericidal action of penicillin: new clues to an unsolved mystery. Trends Microbiol. 2000;8(6):274–8. 10838585 10.1016/s0966-842x(00)01762-5

[92] Ponting CP. Proteins of the endoplasmic-reticulum-associated degradation pathway: domain detection and function prediction. Biochem J. 2000;351 Pt 2:527–35. 11023840 PMC1221390

[93] D'Andrea LD, Regan L. TPR proteins: the versatile helix. Trends Biochem Sci. 2003;28(12):655–62. 14659697 10.1016/j.tibs.2003.10.007

[94] Schmitz-Linneweber C, Small I. Pentatricopeptide repeat proteins: a socket set for organelle gene expression. Trends Plant Sci. 2008;13(12):663–70. 10.1016/j.tplants.2008.10.001 19004664

[95] Boch J, Bonas U. Xanthomonas AvrBs3 family-type III effectors: discovery and function. Annu Rev Phytopathol. 2010;48:419–36. 10.1146/annurev-phyto-080508-081936 19400638

[96] Baker M. Gene-editing nucleases. Nat Methods. 2012;9(1):23–6. 22312637 10.1038/nmeth.1807

[97] Mittl PR, Schneider-Brachert W. Sel1-like repeat proteins in signal transduction. Cell Signal. 2007;19(1):20–31. 16870393 10.1016/j.cellsig.2006.05.034

[98] Tobin GJ, Trujillo JD, Bushnell RV, Lin G, Chaudhuri AR, Long J, et al Deceptive imprinting and immune refocusing in vaccine design. Vaccine. 2008;26(49):6189–99. 10.1016/j.vaccine.2008.09.080 18852005

[99] Mittl PR, Luthy L, Reinhardt C, Joller H. Detection of high titers of antibody against Helicobacter cysteine-rich proteins A, B, C, and E in Helicobacter pylori-infected individuals. Clin Diagn Lab Immunol. 2003;10(4):542–5. 12853383 10.1128/CDLI.10.4.542-545.2003 PMC164274

[100] Yao SY, Luo L, Har KJ, Becker A, Ruberg S, Yu GQ, et al Sinorhizobium meliloti ExoR and ExoS proteins regulate both succinoglycan and flagellum production. J Bacteriol. 2004;186(18):6042–9. 15342573 10.1128/JB.186.18.6042-6049.2004 PMC515170

[101] Fujishige NA, Kapadia NN, De Hoff PL, Hirsch AM. Investigations of Rhizobium biofilm formation. FEMS Microbiol Ecol. 2006;56(2):195–206. 16629750 10.1111/j.1574-6941.2005.00044.x

[102] Wells DH, Chen EJ, Fisher RF, Long SR. ExoR is genetically coupled to the ExoS-ChvI two-component system and located in the periplasm of Sinorhizobium meliloti. Mol Microbiol. 2007;64(3):647–64. 17462014 10.1111/j.1365-2958.2007.05680.x

[103] Chen EJ, Sabio EA, Long SR. The periplasmic regulator ExoR inhibits ExoS/ChvI two-component signaling in Sinorhizobium meliloti. Mol Microbiol. 2008;69(5):1290–1303. 10.1111/j.1365-2958.2008.06362.x 18631237 PMC2652646

[104] Unden G, Becker S, Bongaerts J, Holighaus G, Schirawski J, Six S. O2-sensing and O2-dependent gene regulation in facultatively anaerobic bacteria. Archives of microbiology. 1995;164(2):81–90. 8588737

[105] Liu X, De Wulf P. Probing the ArcA-P modulon of Escherichia coli by whole genome transcriptional analysis and sequence recognition profiling. The Journal of biological chemistry. 2004;279(13):12588–97. 14711822 10.1074/jbc.M313454200

[106] Ravcheev DA, Rakhmaninova AB, Mironov AA, Gel'fand MS. [Comparative genomics analysis of nitrate and nitrite respiration in gamma proteobacteria]. Mol Biol (Mosk). 2005;39(5):832–46. 16240717

[107] Ravcheev DA, Gerasimova AV, Mironov AA, Gelfand MS. Comparative genomic analysis of regulation of anaerobic respiration in ten genomes from three families of gamma-proteobacteria (Enterobacteriaceae, Pasteurellaceae, Vibrionaceae). BMC Genomics. 2007;8:54 17313674 10.1186/1471-2164-8-54 PMC1805755

[108] von Ohle C, Gieseke A, Nistico L, Decker EM, DeBeer D, Stoodley P. Real-time microsensor measurement of local metabolic activities in ex vivo dental biofilms exposed to sucrose and treated with chlorhexidine. Appl Environ Microbiol. 2010;76(7):2326–34. 10.1128/AEM.02090-09 20118374 PMC2849229

[109] Sievers F, Wilm A, Dineen D, Gibson TJ, Karplus K, Li W, et al Fast, scalable generation of high-quality protein multiple sequence alignments using Clustal Omega. Mol Syst Biol. 2011;7:539 10.1038/msb.2011.75 21988835 PMC3261699

[110] Petersen TN, Brunak S, von Heijne G, Nielsen H. SignalP 4.0: discriminating signal peptides from transmembrane regions. Nat Methods. 2011;8(10):785–6. 10.1038/nmeth.1701 21959131

[111] Stamatakis A. RAxML version 8: a tool for phylogenetic analysis and post-analysis of large phylogenies. Bioinformatics. 2014;30(9):1312–3. 10.1093/bioinformatics/btu033 24451623 PMC3998144

[112] Letunic I, Bork P. Interactive Tree Of Life (iTOL): an online tool for phylogenetic tree display and annotation. Bioinformatics. 2007;23(1):127–8. 17050570 10.1093/bioinformatics/btl529

[113] Letunic I, Bork P. Interactive Tree Of Life v2: online annotation and display of phylogenetic trees made easy. Nucleic Acids Res. 2011;39(Web Server issue):W475–8. 10.1093/nar/gkr201 21470960 PMC3125724

[114] Paradis E, Claude J, Strimmer K. APE: Analyses of Phylogenetics and Evolution in R language. Bioinformatics. 2004;20(2):289–90. 14734327 10.1093/bioinformatics/btg412

[115] Harrison A, Dyer DW, Gillaspy A, Ray WC, Mungur R, Carson MB, et al Genomic sequence of an otitis media isolate of nontypeable Haemophilus influenzae: comparative study with H. influenzae serotype d, strain KW20. J Bacteriol. 2005;187(13):4627–36. 15968074 10.1128/JB.187.13.4627-4636.2005 PMC1151754

[116] Saeed-Kothe A, Yang W, Mills SD. Use of the riboflavin synthase gene (ribC) as a model for development of an essential gene disruption and complementation system for Haemophilus influenzae. Appl Environ Microbiol. 2004;70(7):4136–43. 15240293 10.1128/AEM.70.7.4136-4143.2004 PMC444764

[117] Irizarry RA, Hobbs B, Collin F, Beazer-Barclay YD, Antonellis KJ, Scherf U, et al Exploration, normalization, and summaries of high density oligonucleotide array probe level data. Biostatistics. 2003;4(2):249–64. 12925520 10.1093/biostatistics/4.2.249

[118] Bolstad BM, Irizarry RA, Astrand M, Speed TP. A comparison of normalization methods for high density oligonucleotide array data based on variance and bias. Bioinformatics. 2003;19(2):185–93. 12538238 10.1093/bioinformatics/19.2.185

[119] Baldi P, Long AD. A Bayesian framework for the analysis of microarray expression data: regularized t -test and statistical inferences of gene changes. Bioinformatics. 2001;17(6):509–19. 11395427 10.1093/bioinformatics/17.6.509

[120] Tusher VG, Tibshirani R, Chu G. Significance analysis of microarrays applied to the ionizing radiation response. Proceedings of the National Academy of Sciences of the United States of America. 2001;98(9):5116–21. 11309499 10.1073/pnas.091062498 PMC33173

[121] Musser JM, Barenkamp SJ, Granoff DM, Selander RK. Genetic relationships of serologically nontypable and serotype b strains of Haemophilus influenzae. Infect Immun. 1986;52(1):183–91. 3485574 10.1128/iai.52.1.183-191.1986 PMC262217

[122] Farjo RS, Foxman B, Patel MJ, Zhang L, Pettigrew MM, McCoy SI, et al Diversity and sharing of Haemophilus influenzae strains colonizing healthy children attending day-care centers. Pediatr Infect Dis J. 2004;23(1):41–6. 14743045 10.1097/01.inf.0000106981.89572.d1

[123] Shen K, Antalis P, Gladitz J, Sayeed S, Ahmed A, Yu S, et al Identification, distribution, and expression of novel genes in 10 clinical isolates of nontypeable Haemophilus influenzae. Infect Immun. 2005;73(6):3479–91. 15908377 10.1128/IAI.73.6.3479-3491.2005 PMC1111819

[124] Erwin AL, Nelson KL, Mhlanga-Mutangadura T, Bonthuis PJ, Geelhood JL, Morlin G, et al Characterization of genetic and phenotypic diversity of invasive nontypeable Haemophilus influenzae. Infect Immun. 2005;73(9):5853–63. 16113304 10.1128/IAI.73.9.5853-5863.2005 PMC1231076

[125] Barenkamp SJ, Leininger E. Cloning, expression, and DNA sequence analysis of genes encoding nontypeable Haemophilus influenzae high-molecular-weight surface-exposed proteins related to filamentous hemagglutinin of Bordetella pertussis. Infect Immun. 1992;60(4):1302–13. 1548058 10.1128/iai.60.4.1302-1313.1992 PMC256997

[126] Nizet V, Colina KF, Almquist JR, Rubens CE, Smith AL. A virulent nonencapsulated Haemophilus influenzae. J Infect Dis. 1996;173(1):180–6. 8537657 10.1093/infdis/173.1.180

[127] Fleischmann RD, Adams MD, White O, Clayton RA, Kirkness EF, Kerlavage AR, et al Whole-genome random sequencing and assembly of Haemophilus influenzae Rd. Science. 1995;269(5223):496–512. 7542800 10.1126/science.7542800

