# Supplementary figures and images for: Identification and Characterization of msf, a Novel Virulence Factor in Haemophilus influenzae
Source: PLoS One. 2016 Mar 15;11(3):e0149891. doi: 10.1371/journal.pone.0149891 (PMC4792463; doi:10.1371/journal.pone.0149891)

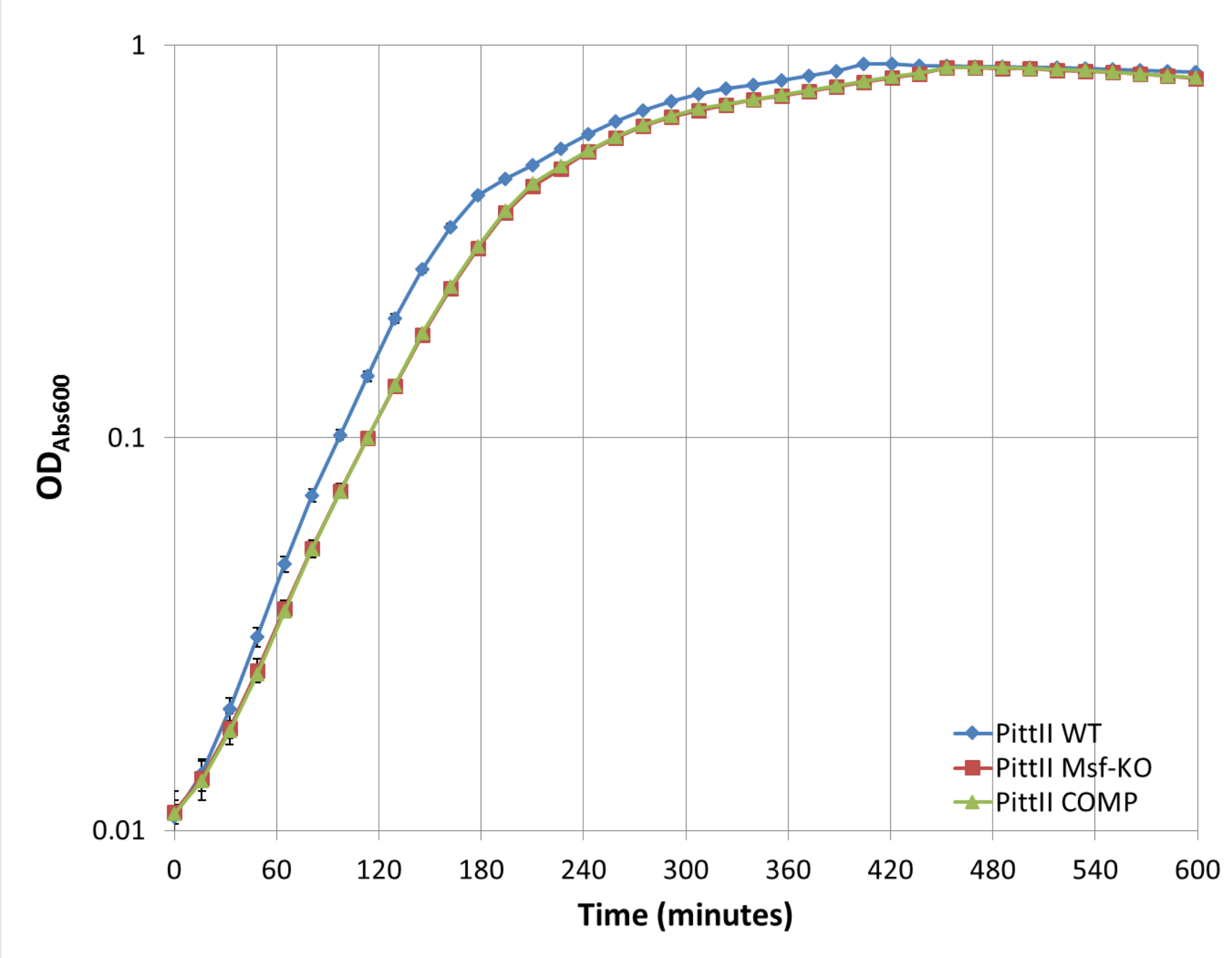

Supplement: S2 Fig — Planktonic growth in BHI broth of 1 mL cultures in 24-well plates. (TIFF) [file pone.0149891.s002.tiff]

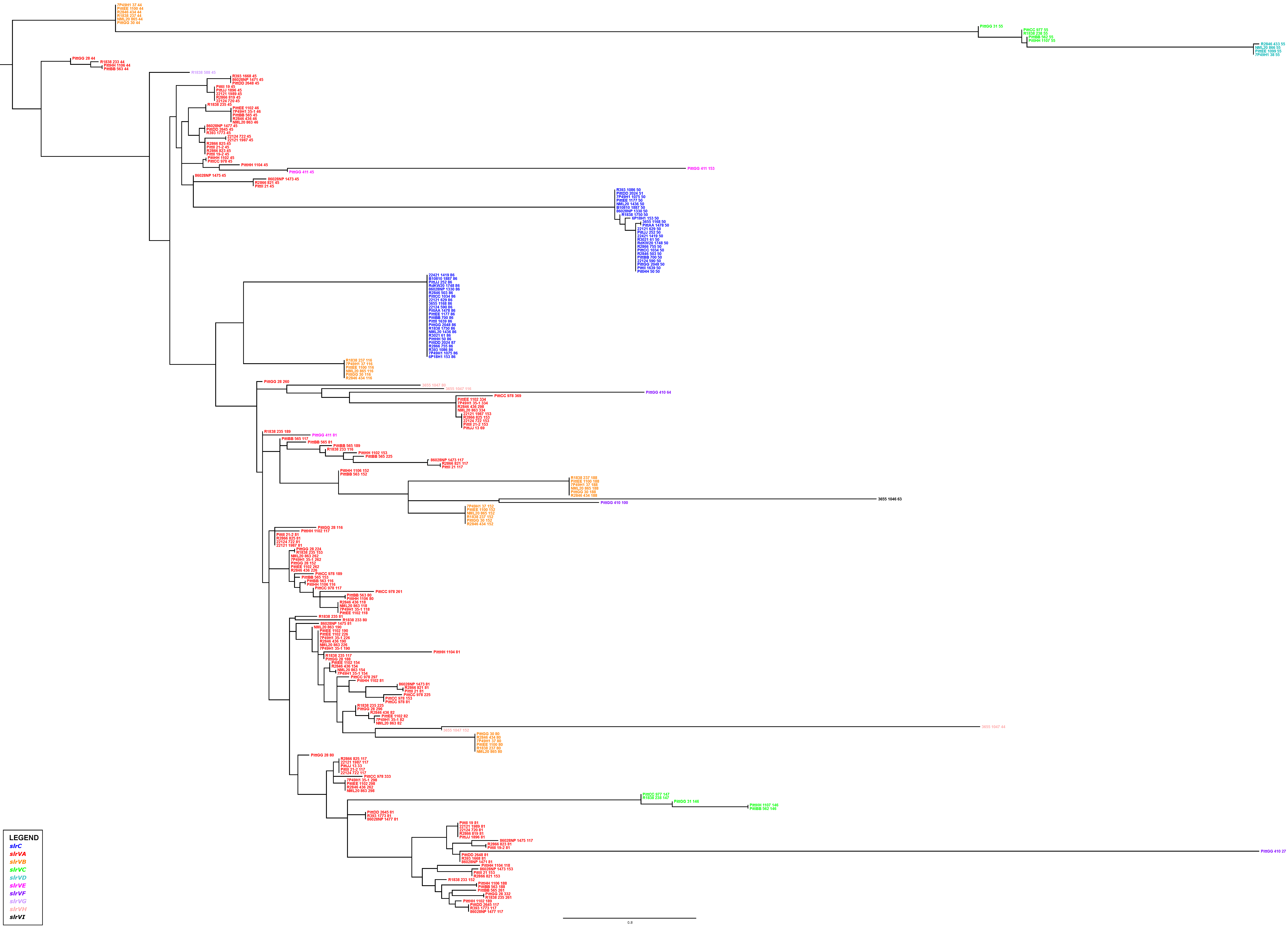

Supplement: S3 Fig — 36 amino acid SLR motifs extracted from the 79 genes containing them (n=256). Motifs found within the same protein do not cluster together. Node labels indicate the strain, strain-specific SGH cluster ID number, and location of the motif within the CDS (amino acid position). This tree corresponds to Fig 3D. (TIFF) [file pone.0149891.s003.tiff]
